# Validation of ART Calculator for Predicting the Number of Metaphase II Oocytes Required for Obtaining at Least One Euploid Blastocyst for Transfer in Couples Undergoing *in vitro* Fertilization/Intracytoplasmic Sperm Injection

**DOI:** 10.3389/fendo.2019.00917

**Published:** 2020-01-24

**Authors:** Sandro C. Esteves, Hakan Yarali, Filippo M. Ubaldi, José F. Carvalho, Fabiola C. Bento, Alberto Vaiarelli, Danilo Cimadomo, İrem Y. Özbek, Mehtap Polat, Gurkan Bozdag, Laura Rienzi, Carlo Alviggi

**Affiliations:** ^1^ANDROFERT, Andrology and Human Reproduction Clinic, Campinas, Brazil; ^2^Faculty of Health, Aarhus University, Aarhus, Denmark; ^3^Anatolia IVF, Ankara, Turkey; ^4^G.E.N.E.R.A., Center for Reproductive Medicine, Rome, Italy; ^5^Statistika Consulting, Campinas, Brazil; ^6^Department of Neuroscience, Reproductive Science and Odontostomatology, University of Naples Federico II, Naples, Italy

**Keywords:** assisted reproductive technology, ART calculator, intracytoplasmic sperm injection, preimplantation genetic testing for aneuploidy, decision support models, POSEIDON criteria, validation study

## Abstract

This multicenter study evaluated the reliability of the recently published ART calculator for predicting the minimum number of metaphase II (MII) oocytes (MIImin) to obtain at least one euploid blastocyst in patients undergoing *in vitro* fertilization/intracytoplasmic sperm injection (IVF/ICSI). We used clinical and embryonic retrospective data of 1,464 consecutive infertile couples who underwent IVF/ICSI with the intention to have preimplantation genetic testing for aneuploidy. The validation procedure followed a stepwise approach. Firstly, we assessed the distribution of euploid blastocysts per patient and found that it followed a negative binomial distribution. Secondly, we used generalized linear models and applied the Lasso procedure–including MII oocytes to adjust the data–to select the factors predicting the response variable “euploid blastocyst.” Third, a logistic regression model–fit to the binomial response euploid (yes/no) for each MII oocyte–was built using the relevant factors. The observational unit was the “woman” whereas the response was the pair (m, n), where n is the number of retrieved MII oocytes and m the corresponding number of euploid blastocysts. The model was internally validated by randomly splitting the data into training and validation sets. The R-squares (~0.25) and the area under the ROC curve (~0.70) did not differ between the training and validation datasets. Fourth, mathematical equations and the calculated probabilities generated by the validation model were used to determine the MIImin required for obtaining at least one euploid blastocyst according to different success probabilities. Lastly, we compared the fittings generated by the validation model and the ART calculator and assessed the predictive value of the latter using the validation dataset. The fittings were sufficiently close for both the estimated probabilities of blastocyst euploid per MII oocyte (*r* = 0.91) and MIImin (*r* = 0.88). The ART calculator positive predictive values, i.e., the frequency of patients with at least one euploid blastocyst among those who achieved the estimated MIImin, were 84.8%, 87.5%, and 90.0% for 70%, 80%, and 90% predicted probabilities of success, respectively. The ART calculator effectively predicts the MIImin needed to achieve at least one euploid blastocyst in individual patients undergoing IVF/ICSI. The prediction tool might be used for counseling and planning IVF/ICSI treatments.

## Introduction

In modern society, the age of the population seeking assisted reproductive technology (ART) is increasing steadily as both women and men tend to postpone childbearing. It is well-known that the female age is the central factor for pregnancy success, with higher ages associated with poorer outcomes (1). However, the frequency of couples with coexistent male infertility has also increased (2, 3). Recent studies have demonstrated that both female age and the etiology and severity of male infertility independently affect reproductive outcomes even under ART settings (4–6).

ART success has been commonly reported as the delivery of a live birth resulting from one initiated or aspirated ART cycle (7). The most comprehensive studies indicate that there is a positive association between the number of retrieved oocytes and live birth rates (LBR), in particular, cumulative LBR, with higher oocyte thresholds for better outcomes (8–10). Although the LBR is the preferable endpoint for couples, it depends on a multitude of controlled and uncontrolled factors, thus making it challenging to use this metric for individualized predictions about the number of oocytes needed to achieve the desired outcome. In 2016, the POSEIDON (**P**atient-**O**riented **S**trategies **E**ncompassing **I**ndividualize**D O**ocyte **N**umber) collaborative group introduced a new metric of success, namely, the ability to obtain the number of oocytes needed to achieve at least one euploid blastocyst for transfer (11–13). Besides the critical role of oocyte numbers on ART success, the transfer of euploid embryos markedly reduces the female age-related decrease in implantation rates (14–16), thus suggesting that the POSEIDON's marker might be a useful endpoint for clinicians providing care to infertility patients.

Recently, a clinical predictive model named “ART Calculator” was developed to estimate the number of metaphase II (MII) oocytes needed to achieve at least one euploid embryo for transfer in each patient undergoing ART (17). The model was built based on clinical and embryonic data of over 300 infertile couples who underwent *in vitro* fertilization/intracytoplasmic sperm injection (IVF/ICSI) and trophectoderm biopsy for preimplantation genetic testing for aneuploidy (PGT-A). The fitted model selected female age, sperm source –adjusted by type of azoospermia whenever appropriate–, and MII oocytes as predictors. A final logistic regression analysis model was developed based on the above predictors to estimate the probability of an MII oocyte become a euploid blastocyst as a function of female age and sperm source. Lastly, an online calculator was created–based on mathematical equations and the probabilities mentioned above–to predict the minimum number of MII oocytes (MIImin) required to obtain at least one euploid blastocyst for specified probabilities of success.

We propose that using pretreatment factors to predict the MIImin could be useful in shared decision-making concerning ART treatments. Herein, we investigated the reliability of the ART calculator using real-world data from couples undergoing ART.

## Materials and Methods

After ethics committee approval, we formed a multicenter collaborative group to enroll consecutive infertile couples who underwent IVF-ICSI treatment intending to have trophectoderm biopsy for PGT-A from July 2017 to August 2018. The ethics committees of *Instituto Investiga*, Campinas, Brazil (CAAE 64291417.0.0000.5599), Hacettepe University, Ankara, Turkey (KA-180069), and Clinica Valle Giulia, Rome, Italy have approved the study.

### Study Population and Patients' Eligibility Criteria

The patients were retrospectively selected using pre-defined inclusion/exclusion criteria from three institutions: Anatolia IVF and Women's Health Center, Ankara, Turkey (Anatolia), G.E.N.E.R.A. center for Reproductive Medicine, Rome, Italy (GENERA), and ANDROFERT, Andrology and Human Reproduction Clinic, Campinas, Brazil (Androfert).

All patients were subjected to IVF/ICSI with the intention to have PGT-A, a test to analyze the DNA of blastocysts for determining genetic abnormalities (aneuploidies). PGT-A was indicated due to advanced maternal age, recurrent miscarriage, repeated implantation failure, severe male factor, and due to patients' concerns about their embryonic ploidy status.

Eligible patients were consecutive infertile couples undergoing their first IVF/ICSI cycle irrespective of the protocol used for ovarian stimulation. We only included patients who reached at least the oocyte pick-up stage, regardless of whether or not a blastocyst was available for biopsy. Moreover, patients were only included if all retrieved MII oocytes were inseminated for own use and the resulting viable blastocysts biopsied. Patients who had PGT for balanced translocations or single-gene diseases were excluded. We also excluded patients treated with donor oocytes, those whose cycles involved injection with both ejaculated and surgically retrieved sperm, and those who used both fresh and frozen-thawed gametes (e.g., fresh and frozen-thawed sperm or fresh and vitrified-warmed oocytes) simultaneously. Cycles in which PGT-A was carried out on vitrified-warmed blastocysts were also excluded.

The participating centers used a unique case report form (CRF) for data collection. Each included couple contributed data concerning only one IVF/ICSI cycle. A total of twenty-three variables were included (Supplementary Table 1). Demographic data included age, body mass index (BMI), infertility duration, infertility factor, antral follicle count (AFC), anti-Müllerian hormone (AMH) levels, and semen parameters. Treatment data comprised the type of ovarian stimulation, gonadotropin regimen, total gonadotropin dose, sperm source for ICSI, and gamete status (fresh or frozen-thawed). Lastly, treatment outcomes included the number of oocytes retrieved, number of MII oocytes retrieved, number of two-pronuclei (2PN) zygotes, number of blastocysts, and number of euploid blastocysts. Codes replaced the records linking patients' identification. Each center's dataset was sent to a third-party statistical company for compilation and analysis.

### Treatment Characteristics

The included couples were evaluated and treated according to each institution's policies, as previously described (18–21). In brief, the ovarian reserve was determined by either AFC or AMH levels, or both, using standardized protocols (22, 23). The AMH values were obtained with the aid of the modified Beckman Coulter generation II assay (23), whereas the AFC was evaluated on the early follicular phase using a two-dimension ultrasound scan (22). Semen analyses were carried out according to the 2010 World Health Organization manual for the examination of human semen (24, 25). The type of azoospermia, when applicable, was determined by the treating physician using a combination of clinical and laboratory data.

The process of ART included ovarian stimulation, oocyte retrieval, fertilization, blastocyst culture, blastocyst biopsy, PGT-A, and subsequent vitrified-warmed embryo transfer. The choice of the ovarian stimulation regimen and gonadotropin dosage was based on the clinician's assessment of ovarian reserve, female age, and history of previous response to ovarian stimulation (18, 19, 26). One of the three protocols was used for ovarian stimulation, namely, (i) long GnRH agonist protocol (Lucrin; Abbott), (ii) GnRH antagonist protocol [Cetrotide (Merck) or Orgalutran (MSD)], and (iii) minimal stimulation protocol. Recombinant FSH [Gonal-F (Merck) or Puregon (MSD)] monotherapy, recombinant FSH combined with recombinant LH [2:1 ratio, Pergoveris (Merck)], recombinant FSH (Gonal-F, Merck) combined with either hMG (Menopur, Ferring) or recombinant LH (Luveris, Merck), or highly purified hMG monotherapy (Menopur; Ferring) were used for ovarian stimulation with initial daily doses varying from 150 to 450 IU. After 5 days of stimulation, the ovarian response was monitored with the use of transvaginal ultrasonography and serum estradiol measurements to adjust daily gonadotropin dosing. Both fixed and flexible GnRH antagonist protocols were used. The antagonist was started on the fifth or sixth day of ovarian stimulation or when the leading follicle achieved 12 mm mean diameter in the fixed and flexible regimens, respectively. The minimal stimulation protocol consisted of either clomiphene citrate or letrozole, followed by a low dose of injectable gonadotropin.

Trigger of final oocyte maturation was achieved by a single subcutaneous injection of (i) recombinant hCG (250 mcg; Ovitrelle, Merck), (ii) urinary hCG (10,000 IU; Gonasi, IBSA), or (iii) GnRH agonist [0.2 mg triptorelin (Decapeptyl; Ferring) or 50 IU buserelin (Suprefact, Sanofi-Aventis)] according to each Center's policies. Oocyte retrieval was carried out under intravenous anesthesia with the use of transvaginal ultrasound-guided puncture of follicles 35–37 h after triggering final oocyte maturation.

### *In vitro* Fertilization Procedures

After 2–4 h of incubation, cumulus-oocyte complexes were denuded by exposure to 80 IU/mL hyaluronidase solution diluted 10-fold with buffered media, and also mechanically by denuding plastic pipettes. Sperm preparation was carried out as previously described (6, 27–29). Insemination of oocytes through ICSI was carried out immediately after denudation (28, 30). Each inseminated oocyte was then placed in a microdroplet of culture medium, covered by pre-equilibrated mineral oil in a micro-well, and loaded into the incubator. Fertilization was checked 16–18 h post-insemination and defined as the presence of two pronuclei (2PN) and two polar bodies. The zygotes were kept in culture to reach the blastocyst stage. Embryo culture was carried out at 37°C under ~6.0% CO_2_ and 5% O_2_ with either a sequential [Quinn's Advantage cleavage-blastocyst media (Origio), G-family media (Vitrolife), and Sidney IVF (Cook)] or a continuous medium [CSCM (Irvine Scientific)], either using a time-lapse (Embryoscope, Vitrolife) or standard incubators (Minc, Cook). Embryo quality was scored according to the criteria described by Gardner (29–31).

### Trophectoderm Biopsy and Preimplantation Genetic Testing

Trophectoderm biopsy was performed on expanding, expanded, and hatched blastocysts (days 5 or 6) (17, 20, 32). In general, zona opening was not performed at the cleavage stage. All biopsies were conducted on a heated stage in a dish prepared with microdroplets of buffered medium overlaid with pre-equilibrated mineral oil. A diode laser was used to assist an opening of 10–15 μm in the zona pellucida (20, 33, 34). Five to ten trophectoderm cells were then aspirated into the trophectoderm biopsy pipette followed by laser-assisted removal of the target cells from the body of the embryo. Biopsied embryos were vitrified.

At Anatolia and Androfert, trophectoderm biopsies were sent to a reference genetic laboratory for the analysis (Genlab, Ankara, and Chromosome, São Paulo, respectively). Samples were processed for whole-genome amplification (WGA) and next-generation sequencing (NGS). In the former, biopsied trophectoderm samples were transferred to 1xPBS solution in PCR tubes, stored at −20°C until 24 samples were collected, and then shipped to the central laboratory. Whole-genome amplification was performed using the Sureplex amplification kit (Illumina, San Diego, CA, USA) (35). After WGA, amplification was checked in gel electrophoresis, and DNA concentration was measured using the dsDNA high-sensitivity assay kit (Qubit® Life Technologies, Waltham, MA, USA). After that, the VeriSeq PGS kit (Illumina, San Diego, CA, USA) was used for NGS library preparation following the manufacturer's protocol for fragmentation, tagmentation, indexing, and purification steps. After normalization, samples were pooled, denatured, and sequenced using Miseq (Illumina, SanDiego, CA, USA). The generated data were analyzed using BlueFuse Multi Software (Illumina, SanDiego, CA, USA). In the latter, the biopsied fragments were immersed into 0.2 mL PCR tubes in a total volume of 2.5 μL of Tris-EDTA buffer pH 8.0 (ThermoFisher Scientific Baltics, Vilnius, Lithuania), frozen at −20 Celsius degrees, and then shipped for analysis. Specimens were subjected to cell lysis, WGA, and construction of libraries using the Ion Reproseq kit (ThermoFisher Scientific, Germany). The DNA quantity was estimated using StepOne (ThermoFisher Scientific, Germany) following the manufacturer's protocol, and NGS was performed using the Ion Torrent PGM™ platform (ThermoFisher Scientific, Germany). Euploidy data analysis was carried out on the Ion Reporter software version 5.2 calibrated at medium sensitivity, using Low-Coverage Whole-Genome workflow (20). At GENERA, the chromosomal analysis was performed through a quantitative real-time polymerase chain reaction (qPCR) as previously described (36). In brief, multiplex amplification of 96 loci (four for each chromosome) was carried out, and a method of relative quantification (37) was applied to predict the copy number status of each chromosome. This comprehensive chromosome testing approach passes through a targeted DNA pre-amplification protocol that does not identify segmental and mosaic aneuploidies (34).

Copy numbers were measured quantitatively, and embryos were classified according to the PGDIS criteria for reporting embryo results (38). In NGS, embryos with <20% of abnormal cells were classified as euploids, whereas embryos with >80% of abnormal cells were deemed aneuploids. Mosaic embryos were those with abnormal cells ranging from 20 and 80%. In qPCR, euploidy was reported when normal chromosomal segregation was detected in each of the 24 chromosomes.

## Statistical Analysis

### Validation Procedure

**Figure 1:**
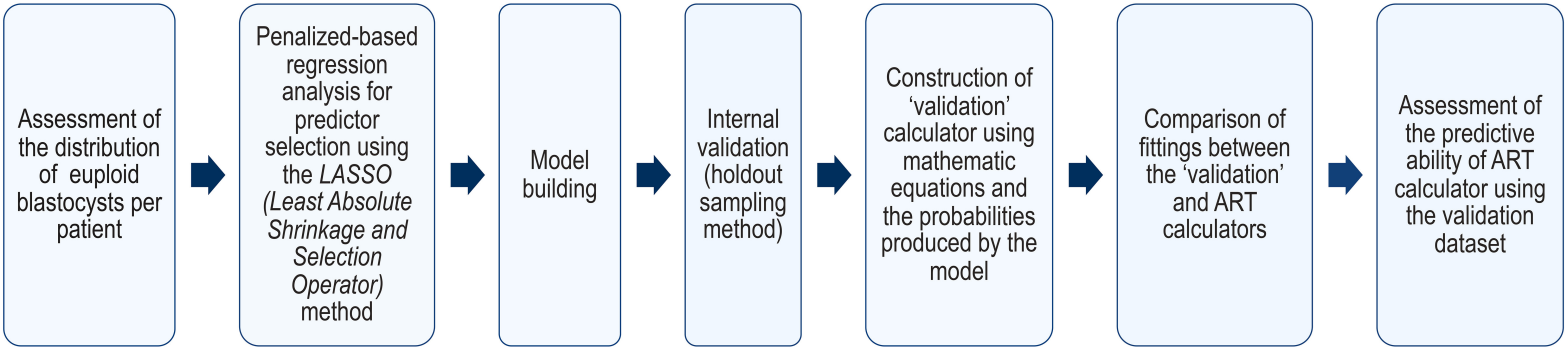
Validation process roadmap.

The validation procedure followed a stepwise approach as depicted in Figure 1.

Firstly, we analyzed the distribution of the number of euploid blastocysts per patient to model the logistic regression analysis. Secondly, we applied a generalized linear model using the adaptive Lasso (Least Absolute Shrinkage and Selection Operator) method, including MII oocytes as a factor to adjust the data, for the selection of predictors concerning the response variable “number of euploid blastocysts” (39, 40). The stopping rule on the Lasso procedure was based on the adjusted Akaike Information Criteria (AIC). We included MII oocytes rather than the total number of retrieved oocytes as the former are the gametes with the capacity to support embryo development to the blastocyst stage and beyond (41, 42). Thus, we avoided the confounding factors that could potentially influence the MII rate. Once the predictors were selected, a logistic regression model–fit to the binomial response euploid (yes/no)–was built. The response was the pair m, N [number of euploid blastocysts (m), number of MII oocytes (N)] for each woman. This logistic model generates the probability (p) as the output, where “p” is the probability that an MII oocyte would turn into a euploid blastocyst, given the relevant predictors. Since participating centers might have different success rates and used distinct genetic analysis platforms, we also included “center” as a predictor to quantify any variation among centers.

The predictive ability of the final model was evaluated by the holdout sampling method. This method randomly split the data into training and validation sets. The training dataset size was 75% of the total, and the validation dataset was 25% of the total. The computation was carried out on the training dataset and its results applied to the validation dataset. Since the validation dataset was not used for the estimations, it can be deemed “new” or “future” data. If the quality of the fits–assessed by the area under the receiver operating characteristics (ROC) curve (AUC)–are comparable between the training and validation datasets, the final model would be apt to be used elsewhere. Then, the probabilities generated by the model were used to determine the MIImin for different success probabilities using the formula MIImin $\geq \frac{\log\left(1-\pi \right)}{\log\left(1-p\right)}$, as previously described (17). The probability of success was denoted by π, and its complement, 1−π, was the risk, i.e., the probability of having no euploid blastocyst despite achieving the estimated MIImin.

Lastly, we compared the fittings generated by the final (validation) model and the ART calculator (17) (https://members.groupposeidon.com/Calculator/) and assessed the predictive value of the latter using the validation dataset. These parameters were the primary validation tests. Graphs and a descriptive correlation measure were used to compare the outputs generated by the validation model and the ART calculator concerning the calculated probabilities “p” and the MIImin. The predictive value of the ART calculator was assessed by computing the frequency of patients with at least one euploid blastocyst among those who achieved the MIImin as predicted by the ART calculator. It is expected that the frequency of cases reaching the MIImin would be at least equal to the probability of success denoted by π. The movie shows how the ART calculator was used to provide the MIImin (see Supplementary Video).

### Sample Size Calculation

The sample size was determined based on the accuracy of the prediction model to estimate the probability “p” that an MII oocyte would turn into a euploid blastocyst (43, 44). For this, we used the ROC curve and set the AUC value as 0.75 and the confidence interval (CI) as 0.07. We estimated *a priori* a 20% loss in the valid cases. Using these assumptions, a dropout inflated sample size of 900 subjects produces a two-sided 95% CI with a width of 0.07 when the AUC is 0.75.

### Missing Data

Data with missing predictor values were excluded *a fortiori* by the regression calculations. Data imputation was not used. Concerning ovarian reserve tests, we included cases in which either the AFC or the AMH value was available.

### Sensitivity Analysis

Since critical embryonic outcomes might impact the estimated probabilities of MII oocytes turning into euploid blastocysts, we assessed whether 2PN fertilization rates and blastulation rates differed among study centers. The 2PN fertilization rate was the number of fertilized oocytes on day 1 (presence of 2PN and two polar bodies assessed at 17 ± 1 h post-ICSI), as a function of all MII oocytes injected. The blastulation rate was the proportion of blastocysts observed on days 5 and 6 post-insemination as a function of the number of 2PN zygotes. The Tukey-Kramer HSD (honestly significant difference) test was used to perform multiple comparisons of means for the variables 2PN fertilization rates and blastulation rates. The test is an exact alpha-level test if the sample sizes are the same, and conservative if the sample sizes are different.

Computations were performed using JMP® PRO 13 (SAS Institute, Cary, North Carolina, US) and PASS 15.0.4 software (NCSS, Kaysville, Utah, USA).

## Results

### Patient Characteristics

**Figure 2:**
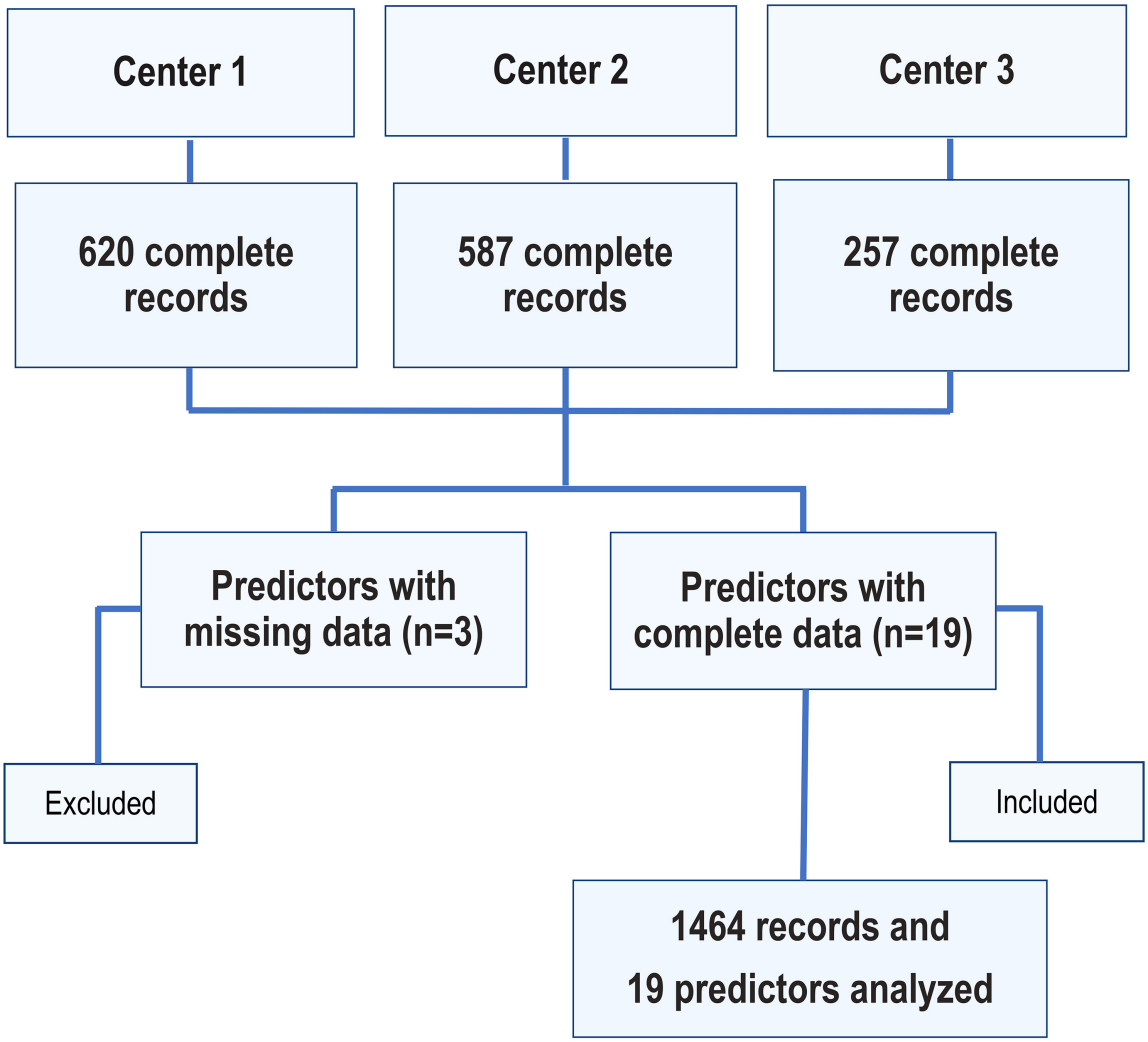
Flowchart depicting the number of included couples and analyzed predictors.

A total of 1,464 patients were included, all of which had a complete IVF/ICSI record for 19 predictors (Figure 2 and Supplementary Table 2). Table 1 shows the distribution of the characteristics of the couples and their IVF/ICSI cycle. The mean female age of our selected cohort was 39.4 years (95% CI: 33.0–44.0 years) with a mean number of MII oocytes retrieved per patient of 6.7 (95% CI: 1.0–16.0). The mean number of blastocysts available for biopsy and PGT-A per patient was 2.1 (95% CI: 0.0–6.0). A total of 9,779 MII oocytes were injected, resulting in 3,108 blastocysts that were subjected to PGT-A. Overall, the percentage of euploid embryos in our cohort was 42.0%. The mean number of euploid blastocysts per patient was 0.9 (95% CI: 0.0–4.0).

**Table 1:** Demographics and treatment characteristics of included couples.

**Characteristics**	***N***	**Median**	**95% CI**
Female age (years)	1,464	39.4	33.0–44.0
Male age (years)	1,464	42.0	33.0–52.0
BMI, female (kg/m^2^)	1,464	24.8	19.0–34.0
BMI, male (kg/m^2^)	333	26.9	21.6–33.1
Infertility factor, N (%)			
Male factor	252 (17.2)	–	–
Unexplained	544 (37.2)	–	–
Endometriosis	58 (3.9)	–	–
Endocrine/Anovulatory	106 (7.2)	–	–
Anatomic/Tubal	55 (3.7)	–	–
>1 type	449 (30.8)	–	–
Baseline FSH (UI/mL)	408	8.6	4.6–14.8
Ovarian reserve marker			
AFC (n)	1,464	9.3	2–22
AMH (ng/mL)	1,287	2.0	0.2–6.8
Semen parameters			
Sperm count (M/mL)	1,464	38.9	0.0–100.0
Total motility (%)	1,395	52.0	20.0–75.0
Sperm morphology (%)	797	3.8	1.0–8.0
DFI (%)	179	21.5	7.0–50.0
Azoospermia, *N* (%)	69 (4.7)		
Non-obstructive; *N* (%)	35	–	–
Obstructive; *N* (%)	34	–	–
Poor ovarian reserve, *N* (%)	482 (32.9)	–	–
Male factor associated (%)	458 (31.3)	–	
Type of ovarian stimulation			
Conventional ovarian stimulation; *N* (%):	1,366 (93.3)	–	–
Minimal stimulation, *N* (%)	98 (6.7)	–	–
Type of gonadotropin; *N* (%)			
rFSH monotherapy		513 (35.1)	–
rFSH + rLH		296 (20.2)	–
rFSH + hMG		538 (36.7)	–
hMG alone		97 (6.6)	–
None		20 (1.4)	–
Total gonadotropin dose (IU)	1,464	3,060.4	850.0–4,950.0
Sperm source for ICSI; *N* (%)			
Ejaculate	1,358 (92.7)	–	–
Epididymis	17 (1.3)	–	–
Testicle	89 (6.0)	–	–
Ejaculated sperm; *N* (%)			
Homologous; normal	761 (56.1)	–	–
Homologous; abnormal	587 (43.2)	–	–
Heterologous	10 (0.7)	–	–
Gamete status for ICSI; N (%)			
Fresh, sperm [S] + oocyte [O]	1,388 (94.8)	–	–
Cryopreserved [S + O]	0 (0.0)	–	–
Combined, fresh [S] + vitrified-warmed [O]	7 (0.5)	–	–
Combined, frozen-thawed [S] + fresh [O]	69 (4.7)	–	–
Oocyte and embryo parameters	1,464		
No. Oocytes retrieved		8.8	1.0–22.0
No. Mature (MII) oocytes		6.7	1.0–16.0
No. Fertilized oocytes (2PN)		4.8	0.0–12.0
No. Blastocysts		2.1	0.0–6.0
No. Euploid blastocysts		0.9	0.0–4.0

*BMI, body mass index; AFC, antral follicle count; AMH, anti-Müllerian hormone; DFI, Sperm DNA fragmentation index; FSH, follicle stimulating hormone; POR, poor ovarian reserve defined according to the POSEIDON criteria, namely, antral follicle count (AFC) <5 and/or AMH hormone <1.2 ng/mL; 2PN, two pronuclei zygote; MII, metaphase II*.

The number of euploid blastocysts per woman followed a negative binomial (Gamma-Poisson) distribution (Supplementary Figure 1). The patient demographics and cycle characteristics by the study's Center are provided in Supplementary Tables 3–5. A total of 620, 587, and 257 patient records were available for analysis by Anatolia, GENERA, and Androfert, respectively. Among the included patients, 19 (1.3%) and 355 (24.2%) had no retrieved metaphase II oocytes and blastocysts available for PGT-A, respectively.

### Development of Validation Model

For the selection of predictors, the stopping rule on the Lasso procedure was based on the adjusted Akaike Information Criteria. The model is a generalized linear model, and the response is the number of euploid blastocysts. The negative binomial distribution was applied to the fit. Accordingly, the link function is the logarithm. For the overdispersion, we chose the identity as the link function. Among the 19 eligible pretreatment predictors (see Supplementary Table 2), the model selected only female age (Supplementary Table 6).

In the validation dataset, however, the number of cases involving azoospermia was small, in particular, when assessing the dataset of Anatolia and GENERA. Given the importance of sperm source in the ART calculator (17), which was highly dependent on the female age, we included sperm source in the final model. Furthermore, owing to the different methods for assessing blastocyst euploid between GENERA (qPCR) and Anatolia/Androfert (NGS), we also included the “study center” in the final model.

Table 2 shows the final fitted predictive model of the binomial response euploid (yes/no) for each MII oocyte using female age, sperm source, and study center, all of which were found to be statistically relevant predictors. In particular, sperm source only applied to the comparisons between ejaculated sperm and testicular sperm from men with NOA. Moreover, the effect of the “study center” was exclusively noted when the Italian center was compared to the two other centers. Supplementary Table 7 shows the previously published ART calculator predictive model for comparison purposes only. In the latter, only the female age and sperm source were relevant predictions. Notably, the original ART calculator model was developed using a single-center dataset, thus making the “study center” irrelevant for model comparison.

**Table 2:** Final validation model for prediction of the probability (p) of euploid blastocyst per mature (MII) oocyte.

*Y* = −2.728414 –0.138868 [I(spermSource=Ejaculate) – I(spermSource=Testicular_NOA)] −0.13032 [I(spermSource=Testicular_NOA) – I(spermSource=NOA)] +0.4928267[I(spermSource = Ejaculate) – I(spermSource=Testicular NOA)] +0.0807783[I(Center = Anatolia) – I(Center = Androfert)] +0.3765617[I(Center = GENERA) – I(Center = Anatolia)] *Where the indicator function I(x) = 1 if x is TRUE and 0 otherwise. The probability p is* $p=\left(\frac{1}{1+e^{-Y}}\right)$						
**Term**	**Estimate**	**Std error**	**Wald ChiSquare**	**Prob** **>** **ChiSquare**	**Lower 95%**	**Upper 95%**
Intercept (a)	−2.728414	0.183	220.281	<0.0001	−3.088	−2.368
spermSource[Ejaculate-Testicular_NOA]:(ageFemale-39.414)	−0.138868	0.007	306.601	<0.0001	−0.154	−0.123
spermSource[Testicular_NOA-NOA]:(ageFemale-39.414)	−0.13032	0.027	22.471	<0.0001	−0.184	−0.076
spermSource[Ejaculate-Testicular NOA]	0.4928267	0.179	7.570	0.006	0.141	0.843
Center [Anatolia-Androfert]	0.0807783	0.105	0.591	0.441	−0.125	0.286
Center [GENERA-Anatolia]	0.3765617	0.068	30.010	<0.0001	0.241	0.511
Response: euploid blastocyst per MII oocytes			–LogLikelihood: 1527.242			
Distribution: negative binomial			Number of Parameters: 6			
Estimation method: adaptive Lasso			BIC: 3,098.065			
Validation method: AICc			AICc: 3,066.544			
Probability model link: Logit			Generalized RSquare: 0.258			
Number of rows: 1,464			Area under the curve: 0.700			

*The full equation is written at the top of the table. Each particular characteristic is displayed with an associated P-value (Prob >ChiSquare) giving the indication of how much weight each variable will contribute to the probability of blastocyst euploidy per metaphase II oocytes*.

**Figure 3:**
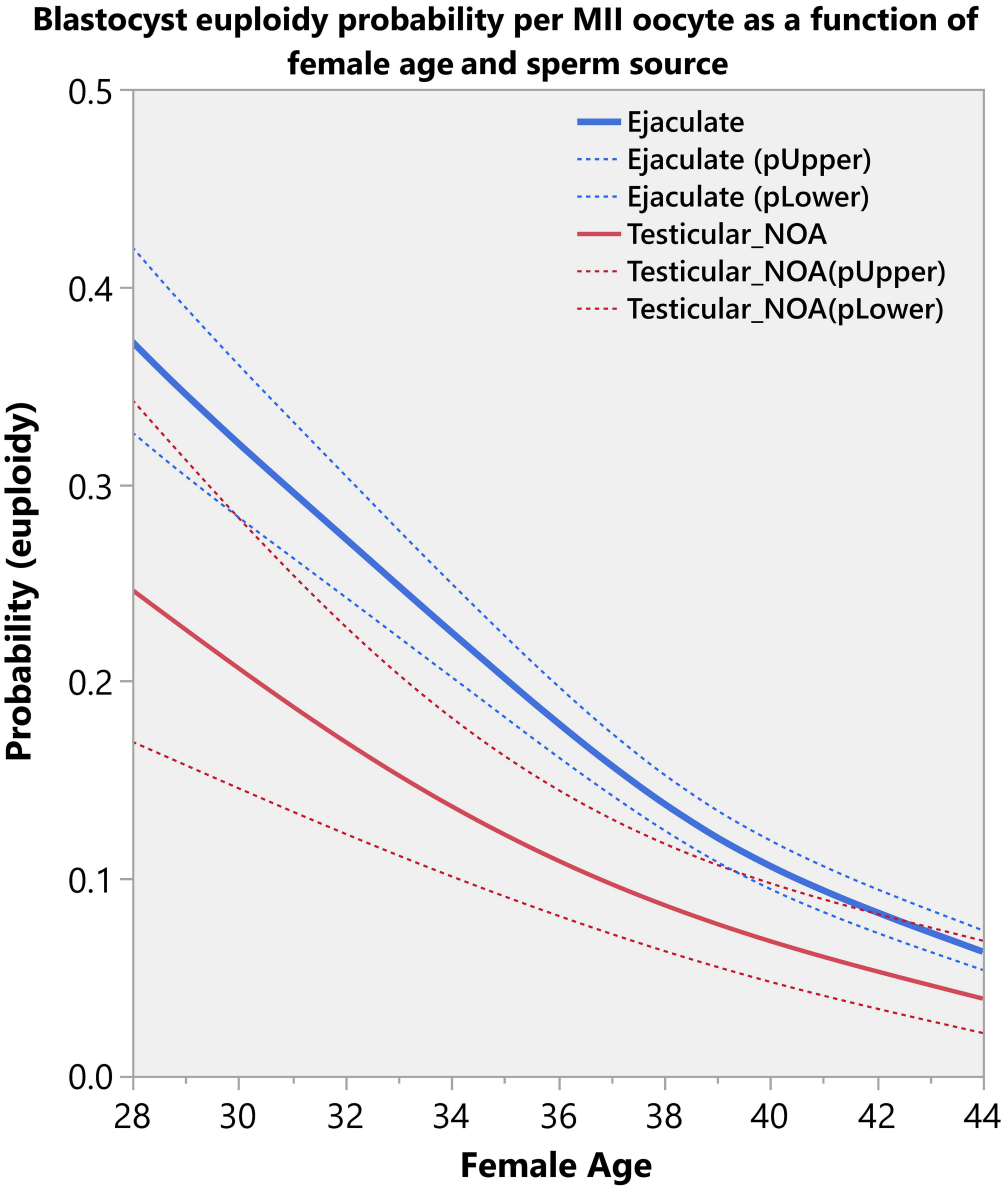
Blastocyst euploidy probability per mature (MII) oocyte. The plots show the probability of an MII oocyte turn into a euploid blastocyst as a function of female age and sperm source. The estimated probabilities (solid curves) and their 95% CI (dotted curves) are presented according to sperm source for IVF/ICSI, namely, ejaculated sperm (blue) and testicular sperm extracted from patients with non-obstructive azoospermia (NOA) (red). The relations are non-linear and characterized by a differential modulatory effect of sperm source across age (see text).

Figure 3 shows the predicted probabilities of an MII oocyte turning into a euploid blastocyst, which decreased progressively as a function of the female age. Overall, the probabilities were negatively modulated by the use of testicular sperm from men with NOA across age. The effect of sperm source was highly dependent on the female age.

**Figure 4:**
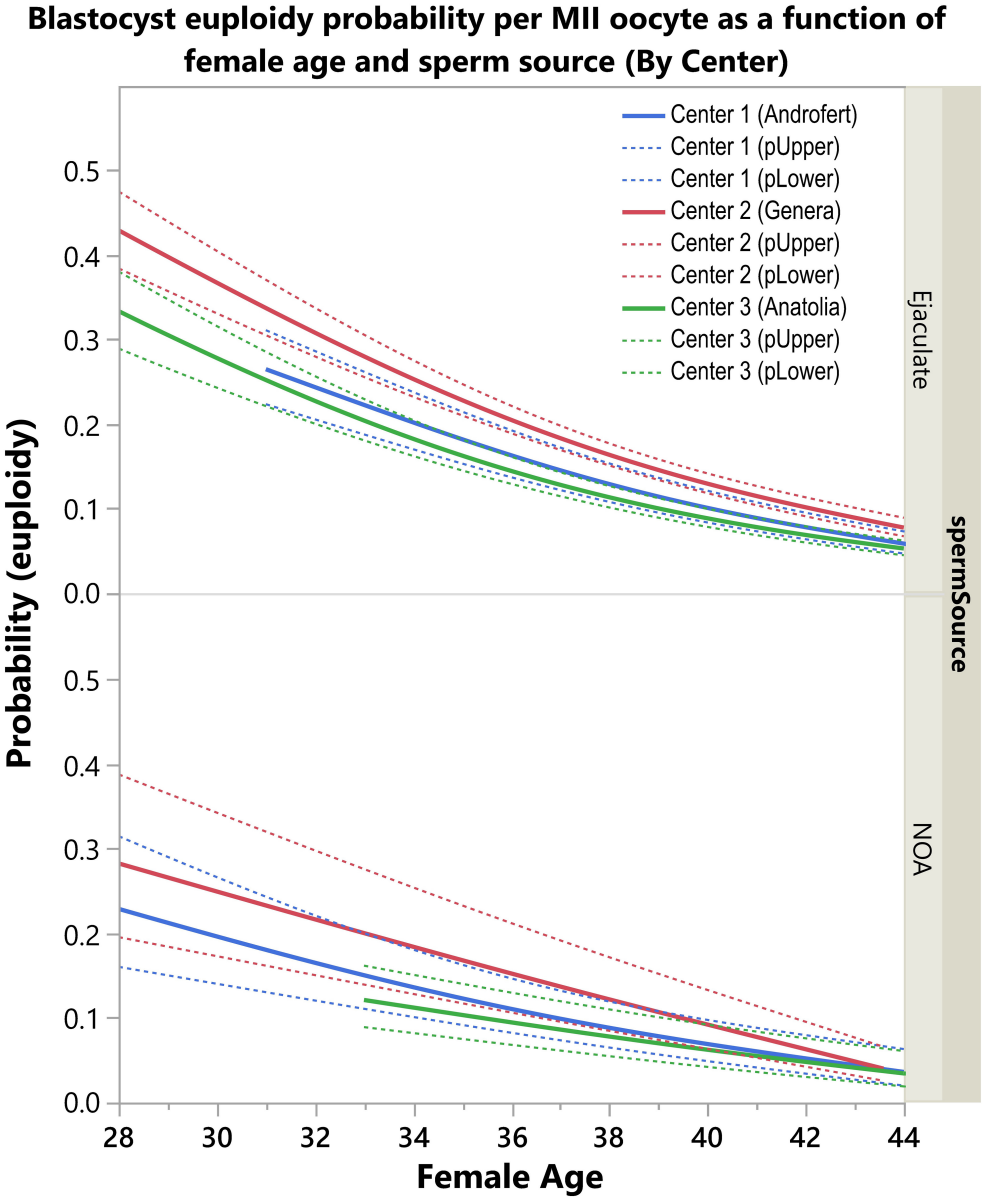
Center effect (genetic analysis method) on blastocyst euploidy probability per mature (MII) oocyte. The plots show the probability of an MII oocyte turn into a euploid blastocyst as a function of female age and sperm source by Center. The estimated probabilities (solid curves) and their 95% confidence interval (dotted curves) are presented according to sperm source for IVF/ICSI, namely, ejaculated sperm (top) and testicular sperm extracted from patients with non-obstructive azoospermia (NOA) (bottom). Centers 1 and 3 utilized next generation sequencing (NGS) for the analysis of trophectoderm biopsies whereas Center 2 used quantitative real-time polymerase chain reaction (qPCR). The relations are non-linear and characterized by a differential modulatory effect of sperm source and genetic analysis method across age (see text).

Figure 4 shows the relative influence of the “study center” on the calculated probabilities. The figure shows the probabilities according to female age and sperm source. The fittings revealed that the probability of an MII oocyte turning into a euploid blastocyst was overall impacted by the study center. Notably, the fittings were very close between the Turkish and Brazilian centers. Both centers used NGS for blastocyst chromosomal analysis, which coincides with the platform utilized to construct the ART calculator model. By contrast, the probabilities of an MII oocyte turning into a euploid blastocyst were higher in the Italian center than the Turkish and Brazilian centers. The former analyzed the blastocysts through qPCR comprehensive genetic screening.

### Sensitivity Analysis

Analysis of embryonic outcomes that might have influenced the estimated probabilities of an MII oocyte to turn into a euploid blastocyst demonstrated that the means concerning 2PN fertilization rates and blastulation rates were not significantly different among study centers (Supplementary Data Sheet).

### Assessing Ability to Predict Blastocyst Euploidy Probability

**Figure 5:**
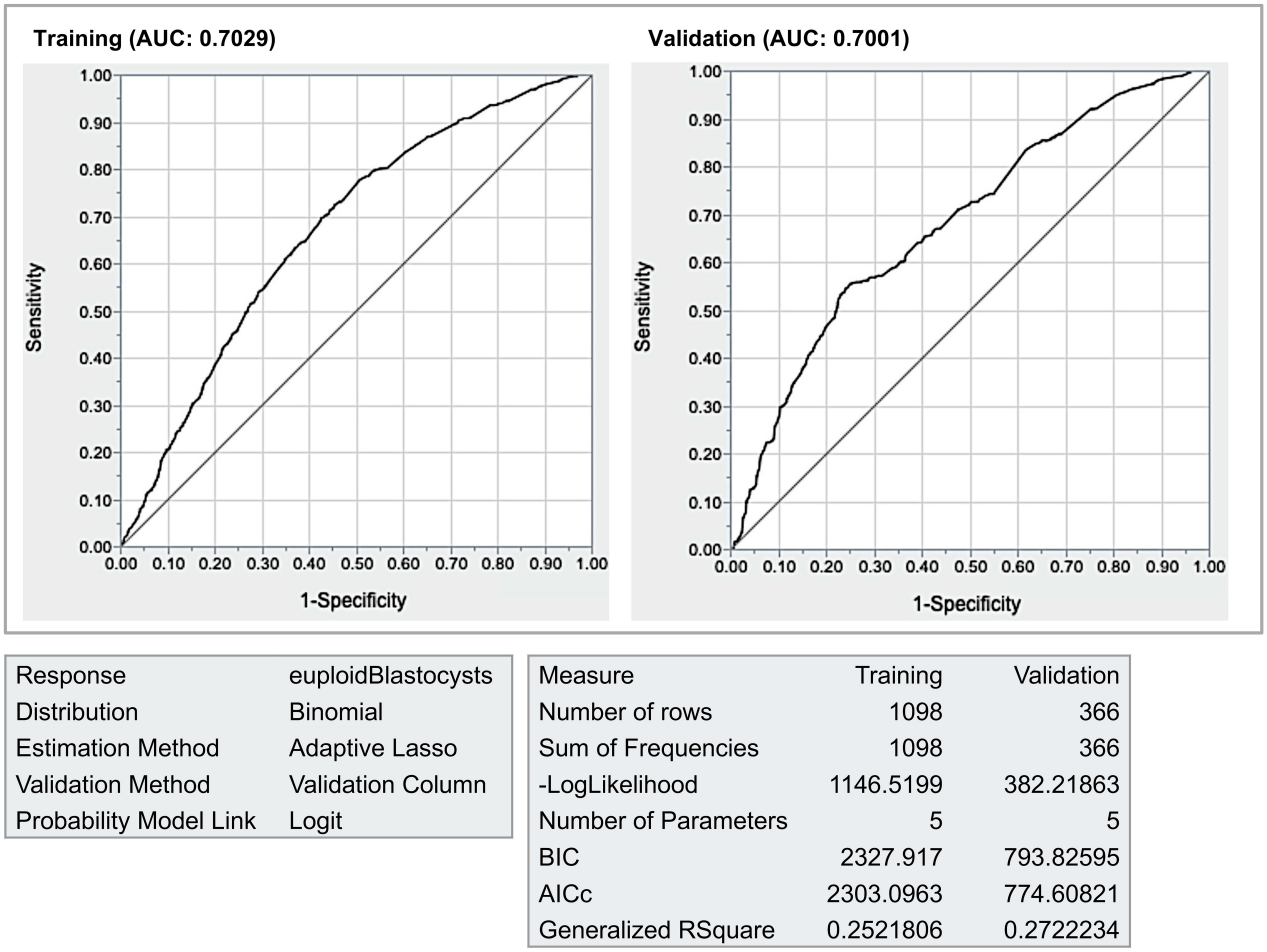
Internal validation. The final model was validated by the holdout method (75% of the data in the training dataset, 25% on the validation data set). The areas under the receiver operating characteristics curves (AUC) curves (~0.70) and the generalized RSquare results (~0.26) were similar, thus indicating that the results should hold true for future data.

The internal validation by the holdout sampling method revealed that both the R-squares (~0.26) were very close between the validation and training datasets. Moreover, both the AUC (~0.70) and the ROC curves were also practically identical (Figure 5).

### Comparison of Fittings

**Figure 6:**
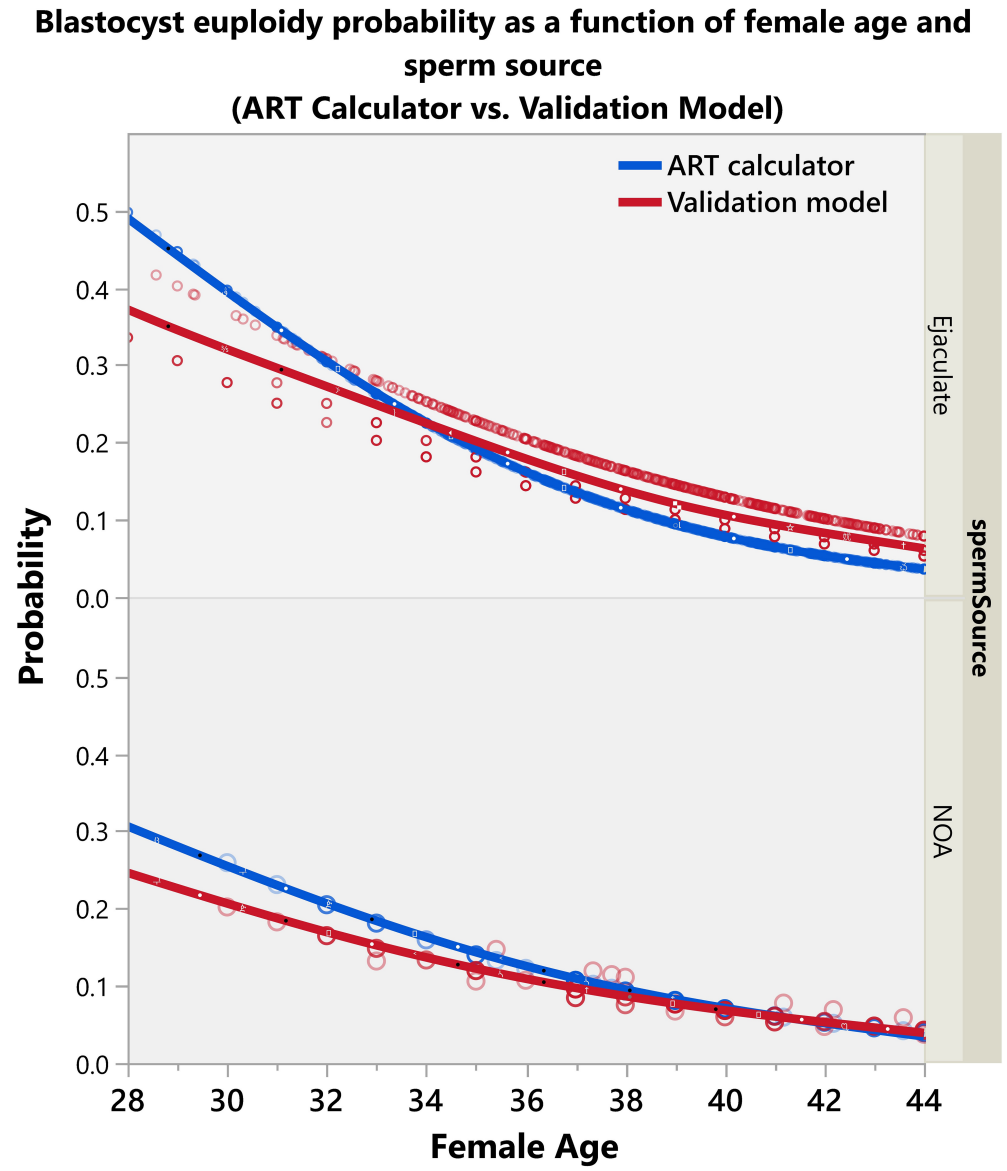
Plots showing the predicted blastocyst euploidy probabilities (per MII oocyte) by the validation model and ART calculator.

Figure 6 shows the comparison of predicted blastocyst euploidy probabilities per MII oocyte between the validation model and ART calculator. The curves depict the probabilities according to the female age and sperm source. Both age and type of sperm used for ICSI were influential; younger women and the use of ejaculated sperm for ICSI were associated with a higher chance of having a euploid blastocyst per MII oocyte. The fittings generated by the ART calculator and validation model were similar. The median absolute difference in the predicted probabilities between both models was 0.02 (95% CI 0.00–0.05) (Supplementary Figure 2).

Supplementary Figure 3 shows the relative influence of the “study center” for assessing blastocyst euploidy on the calculated probabilities. The fittings of both the Turkish and Brazilian centers were very close to that of the ART calculator, in particular among women of 35 years and older; this subset of patients comprised 94% of the validation dataset (Supplementary Figure 4). Furthermore, the fittings of the Italian center and the ART calculator showed similar shapes. Still, the former yielded slightly higher blastocyst euploidy probability per MII oocyte across age than that of the ART calculator. The mean absolute differences on the predicted probabilities between the ART calculator and validation model by country were 0.011 and 0.015 in the Turkish and Brazilian centers [95% interquartile ranges 0.015 (Androfert) and 0.008 (Anatolia)], respectively, whereas it was 0.047 in the Italian center [95% interquartile range 0.005 (GENERA); Supplementary Figure 5].

**Figure 7:**
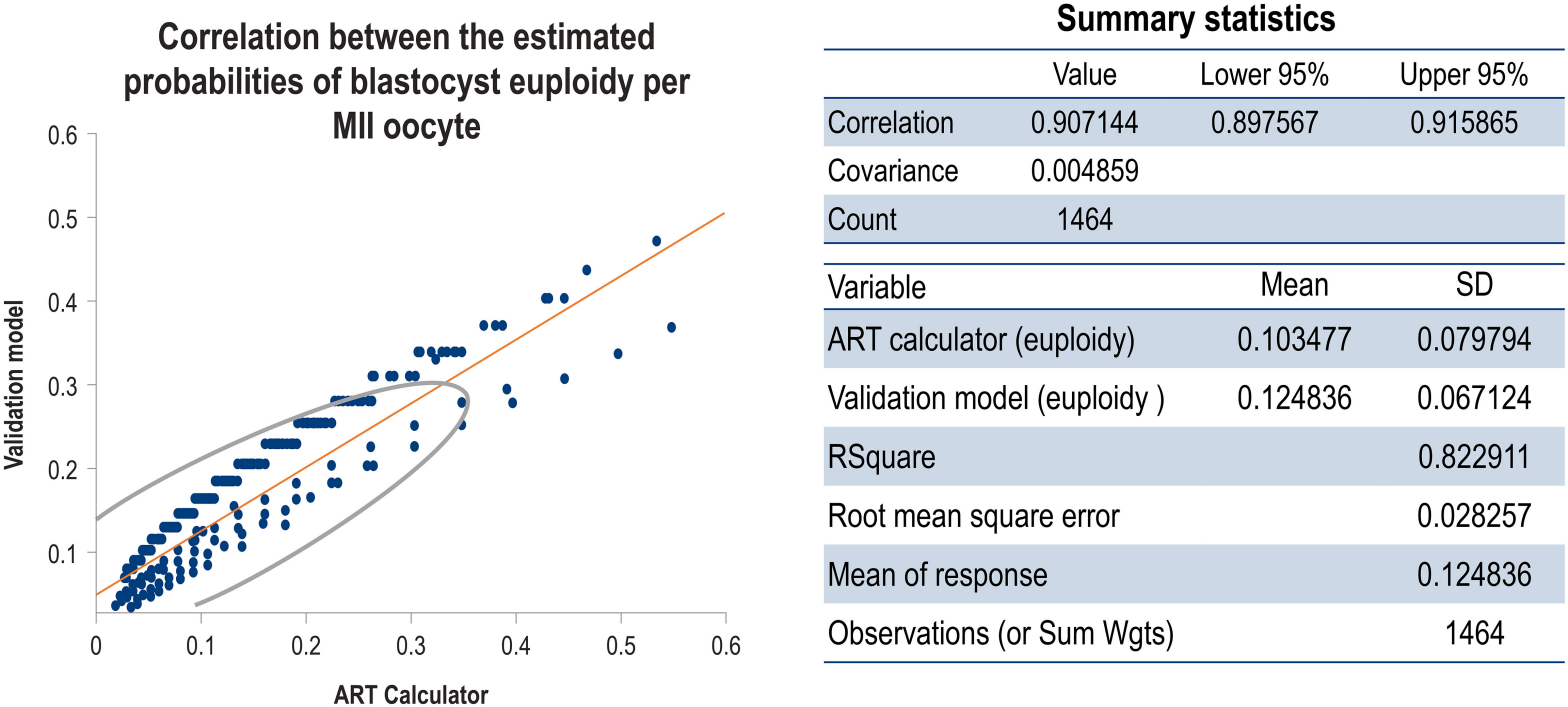
Scatterplot showing the correlation between the ART calculator and validation model concerning the predicted probabilities of blastocyst euploid per MII oocyte. The density ellipse contains 95% of the points.

**Figure 8:**
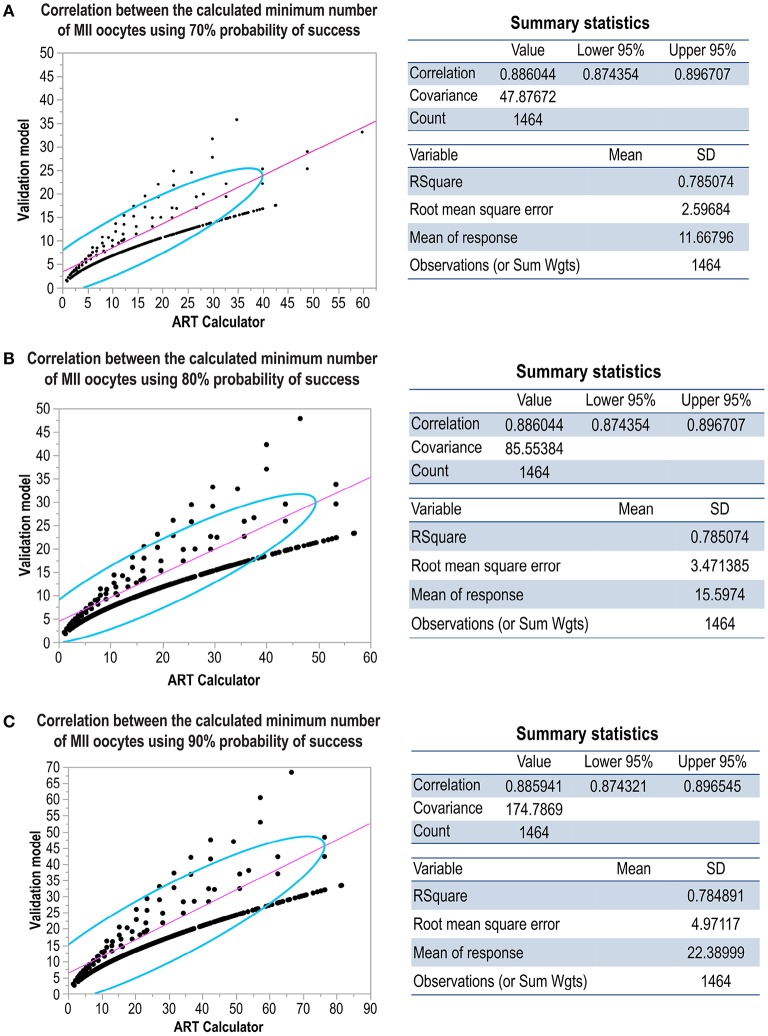
Scatterplots showing the correlation between the ART calculator and validation model concerning the predicted minimum number of MII oocytes required for achieving at least one euploid blastocyst with user-defined 70% **(A)**, 80% **(B)**, and 90% **(C)** probabilities of success. The density ellipse contains 95% of the points.

Figure 7 shows the correlation concerning the predicted probabilities of blastocyst euploid per MII oocyte between the validation model and ART calculator; the probabilities were highly correlated (*r* = 0.91). Figure 8 shows the correlation between the MIImin estimated by the ART calculator and the validation model. The formula *MIImin* $\geq \frac{\log\left(1-\pi \right)}{\log\left(1-p\right)}$ was used to compute the minimum number of MII oocytes required to obtain at least one euploid blastocyst. The figure shows the correlations according to three user-defined probabilities of success (π) concerning the estimations, namely, 70% (Figure 8A), 80% (Figure 8B), and 90% (Figure 8C). In all scenarios, the MIImin estimated by the ART calculator was highly correlated with the MIImin estimated by the validation model (*r* ~ 0.88).

### ART Calculator Predictive Ability

The validation dataset comprised of 1,464 patients was used to assess the ART calculator performance. The frequencies of patients with at least one euploid blastocyst among those who achieved the predicted MIImin by the ART calculator (positive predictive value) were 84.8, 87.5, and 90.0% for 70, 80, and 90% probabilities of success (π), respectively (Table 3).

**Table 3:** ART calculator predictive value.

			**At least one euploid blastocyst**				
			**Yes (*N*)**	**%**	**No (*N*)**	**%**	**Total (*N*)**
ART Calculator	Probability of success (π)						
	70%	MIImin (=yes)	317	**84.8%**	57	15.2%	374
		MIImin (=no)	334	30.6%	756	69.4%	1090
	80%	MIImin (=yes)	217	**87.5%**	31	12.5%	248
		MIImin (=no)	434	35.7%	782	64.3%	1216
	90%	MIImin (=yes)	135	**90.0%**	15	10.0%	150
		MIImin (=no)	516	39.3%	798	60.7%	1314

*The validation dataset (N = 1,464 patients) was used to compute the frequencies of patients with at least one euploid blastocyst among those who achieved the predicted minimum number of metaphase II oocytes (MIImin) according to the ART calculator (positive predictive value; PPV) for three probabilities of success. The PPV are highlighted in bold*.

## Discussion

We have validated a point-of-care clinical tool, named “ART calculator,” to assist clinicians in predicting the minimum number of MII oocytes required to achieve at least one euploid blastocyst for transfer in infertile couples undergoing IVF/ICSI through the use of a database obtained from a retrospective analysis of three institutions. The validation procedure followed the same steps applied during the development of the ART calculator (17), but it included an external cohort 5-fold bigger than that used in the latter. The model was reliable and adequately predicted the MIImin for different user-defined probabilities of success. The similarities between the predictive ability of the validation and ART calculator models indicate that the estimations should hold for future data. While the ART calculator performed better when NGS was the method for blastocyst chromosome screening, it also correlated well with qPCR data.

The clinicians counseling infertile couples who are embarking on ART may now have an additional tool to provide individualized recommendations regarding the MIImin required to achieve at least one euploid blastocyst for transfer. Personalized tools to objectively assess the probability of success in ART are urgently needed because patients do not fully understand the association between the availability of oocytes and embryos and pregnancy failure. Thus, proper counseling regarding the chances of success in ART needs improvement. The availability of at least one euploid embryo for transfer has a major impact for the patient undergoing ART, as ~50–60% of euploid blastocysts implant across all age categories (15, 16). The ART calculator may help to discuss these issues by providing an objective assessment of the number of oocytes needed to optimize the chances of implantation, with potential clinical utility for guidance concerning the development of a workable therapeutic plan to reduce the time to live birth.

### Interpretation

The ART calculator focuses primarily on pretreatment predictors, in particular, female age and type of sperm used for IVF/ICSI, to assist with the informed decision-making process. In this study, we confirmed the role of the female age by assessing a large validation dataset of three ART centers from three countries. Importantly, our dataset included consecutive infertile couples undergoing IVF/ICSI with the intention of having PGT-A. It means patients were included irrespective of having a blastocyst available for biopsy, likewise in the ART calculator original study (17). This feature of the study's design was essential to accurately estimate the number of MII oocytes required to achieve at least one euploid blastocyst because many patients undergoing ART do not have either MII oocytes retrieved or embryos available for PGT-A.

Firstly, we analyzed the distribution of the number of euploid blastocysts per couple and found that it followed a negative binomial (Gamma-Poisson) distribution. This distribution was the same attained in the ART calculator original dataset, thus confirming previous observations (17). Then, we assumed the negative binomial model for the number of euploid blastocysts, and applied a penalized method, named the Lasso, for variable selection (39, 40). The negative binomial was chosen from the first principles and from the heuristic fact that this distribution fitted the data very closely. The method, which allows for the fitting of correlated and high-dimensional data (39, 40, 45), removed redundant variables and selected female age as the only relevant predictor.

Subsequently, we built a generalized regression model–fit to the binomial response euploid (yes/no) for each MII oocyte–using predictors deemed relevant. The response was the pair m, N [number of euploid blastocysts (m), number of MII oocytes (N)] for each woman. In addition to the female age, we included “sperm source” in the final fitted model for two reasons. Firstly, it was deemed necessary in the ART calculator development study. Secondly, the number of cases involving non-ejaculated sperm was small in the validation dataset, which might have resulted in the removal of this predictor by the LASSO method.

Moreover, we included the technique of blastocyst euploidy assessment as they differed between the study centers. The Italian center utilized qPCR, whereas the Turkish and Brazilian centers applied NGS. Unlike NGS, qPCR does not highlight embryos with a PGT-A result falling in the mosaic range (46).

Indeed, the validation model confirmed that the effect of sperm source was highly dependent on the female age, thus confirming the results of the ART calculator study (17). Our data indicate that the estimated probability of an MII oocyte turn into a euploid blastocyst decreases progressively with female age, an effect that is negatively modulated by the use of testicular sperm from men with NOA, like that observed in the ART calculator development study. While the impact of testicular sperm was meaningful in younger women, it was practically offset in women of 40 years and over, thus indicating that the effect of advanced female age on embryo quality is so dramatic that it cannot be changed significantly by any other factor. Of note, these results must be interpreted with caution given the limited number of men with azoospermia and women younger than 35 years in our dataset. The blastocyst euploidy probabilities–as shown in Figure 3–are more meaningful for the female age range between 35 and 44 years and ICSI cases involving the use of ejaculated sperm, which comprised over 95% of our dataset.

Nevertheless, our data are consistent with previous reports, which showed that the use of testicular sperm from men with NOA adversely affects the likelihood of obtaining a euploid blastocyst per oocyte pickup. This effect is caused primarily by the lower fertilization rate and blastocyst development rate with the use of testicular sperm than ejaculated sperm (6, 17, 47). Thus, the sperm source has to be discussed in certain situations, although the most critical factor in predicting the number of mature oocytes for at least one euploid blastocyst is the female age. With aging, oocyte chromosomal abnormalities and cytoplasmic dysfunctions are increased, whereas the number of primordial follicles progressively declines (20, 48–50). Consequently, both embryo quantity and quality are reduced, thus explaining the reasons why IVF success is lower in older women than in younger counterparts (51).

The validation model revealed that the probability of blastocyst euploidy per MII oocyte was affected by the center in which IVF/ICSI was carried out. Since participating centers might have different success rates, we assessed whether critical embryonic outcomes impacted the blastocyst euploidy probability. We found that there were no differences in 2PN fertilization and blastulation rates among centers (Supplementary Data Sheet). These findings suggest that the genetic analysis method was the likely reason explaining the differential blastocyst euploidy probability per MII oocyte between the Italian and Turkish/Brazilian centers. As previously mentioned, the genetic analysis platform used to construct the ART calculator was the same as the one used by the Turkish and Brazilian centers. As expected, the mean absolute difference in the predicted probabilities between the ART calculator model and the validation model using the Turkish and Brazilian centers combined was very low (1%). By contrast, the probabilities of an MII oocyte turn into a euploid blastocyst were higher in the Italian center than the Turkish and Brazilian centers. The former analyzed the blastocysts through quantitative real-time polymerase chain reaction (qPCR) comprehensive genetic screening. The higher blastocyst euploidy probabilities per MII oocytes in GENERA relates to the fact that allegedly mosaic embryos are not reported. At GENERA, the decision of not reporting mosaic embryos relies on the current limitations of diagnosing chromosomal mosaicism from a single trophectoderm biopsy rather than to the molecular technique (52–55). Although the effect of “study center”–and its inherent differences concerning the type of utilized genetic analysis–was statistically significant, its clinical impact seems to be less relevant. Indeed, the mean absolute difference in the predicted probabilities generated by the ART calculator model and validation model was still low (4%) when only the Italian center was considered.

The next steps of our validation study were essentially mathematical. We assessed the prediction ability of the validation model by the holdout sampling method, which randomly splits the data in two, known as training and validation datasets. The quality of the predictive model—assessed by comparing the ROC curves between the training and validation (holdout) datasets—was similar to that of the ART Calculator (0.70 vs. 0.72, respectively), thus suggesting that both models can be used elsewhere. For predictive models, calibration using an external dataset might increase performance owing to the homogeneity of the studied population (56). However, in our study, the calibration of the ART calculator using the external (validation) dataset did not improve its performance. In both models, the infertile couple was the observational unit and the pair (m, n), the response (where “n” is the number of metaphase II oocytes and “m” the corresponding number of euploid blastocysts). A heterogeneous (mixed) Poisson model might have produced the negative binomial distribution for the number of euploid blastocysts. The heterogeneity is expected given the distinct women ages. Thus, given the observations above and the complex nature of the process in which an MII oocyte might end up into a euploid blastocyst, the original ART calculator model with a ~72% predictive ability should be the one to be used clinically.

Importantly, the objective of the validation model—as well as the ART calculator—was to develop a prediction formula for estimating the minimum number of MII oocytes needed to achieve at least one euploid blastocyst. There was no attempt to determine fundamental associations between the predictors and blastocyst euploidy (57). Thus, other known and unknown predictors might also influence blastocyst euploidy, but the inclusion of additional predictors from the existing dataset did not materially affect the estimates.

After the internal validation discussed above, the same model was run with the full dataset, that is, comprising the training and validation datasets, to predict the probability “p” of blastocyst euploidy per MII oocyte. The model itself was logistic, and the derived coefficients defined the linear expression “y” to obtain “p”. The values of “p” were highly correlated between the validation model and the ART calculator (*r* > 0.9). The final endpoint was the MIImin oocytes required to obtain at least one euploid blastocyst. This endpoint was estimated using the value of “p” and the probability of success (i.e., the probability of having at least one euploid blastocyst if the predicted number of MII oocytes is achieved). Again, the MIImin generated by the validation model and ART calculator were highly correlated overall (r ~ 0.9).

Lastly, we assessed the ART calculator's usefulness by computing its positive predictive ability. It was expected that the frequency of couples that achieved the MIImin—as predicted by the ART calculator—and had at least one euploid blastocyst would be at least equal to the user-defined probability of success. Indeed, the positive predictive values were equal or slightly higher than the correspondent user-defined probabilities of success, thus confirming the clinical utility of the predictive tool, which is available online at https://members.groupposeidon.com/Calculator/.

### Clinical Importance

In practical terms, the estimations provided by the ART calculator should be analyzed according to the probability of success, denoted by “π” (e.g., 70%, 80%, 90%), set by the user. Based on the ART calculator, an exemplary patient of 35 years-old embarking on IVF/ICSI, whose male partner is non-azoospermic, requires a total of five (95% CI: 5–6), seven (95% CI: 6–9), and ten (95% CI: 9–13) MII oocytes to obtain at least one euploid blastocyst for 70, 80, and 90% probabilities of success. It means that among couples achieving those figures, the risk, denoted by 1−π, of having no euploid blastocyst despite achieving the predicted MIImin will be, respectively, 30, 20, and 10%. Since each euploid blastocyst has an implantation potential of ~50–60% irrespective of the age group (15, 16, 32), then if all other factors are adequate, the cumulative pregnancy rates among patients who achieve the MIImin as per the calculator estimation should be 50–60% or higher.

The model is primarily intended to be a counseling tool for shaping patients' expectations and preparing them both emotionally and financially for the treatment journey. From a clinical and embryological perspective, the ART calculator outputs might also be used to help clinicians design individualized patient-oriented treatment strategies aiming at obtaining the number of MII oocytes needed for achieving at least one euploid blastocyst for transfer in IVF/ICSI procedures. The provision of such an objective estimation could help the clinician with regards to treatment planning. The individualized oocyte number may be achieved using patient-oriented strategies. For instance, the type of GnRH analog, type of gonadotropin, the starting dose, and the regimen could be tailored accordingly (58–63). A comprehensive review of the patient-oriented strategies encompassing individualized oocyte number can be found in a series of articles compiled in a dedicated Frontiers in Endocrinology research topic (https://www.frontiersin.org/research-topics/6849/poseidons-stratification-of-low-prognosis-patients-in-art-the-why-the-what-and-the-how).

### Strengths and Limitations

To our knowledge, this is the first study to validate the ART calculator using an external patient cohort. The results are clinically significant for all stakeholders, including patients, healthcare providers, and policymakers. The primary use of the model is to serve as a counseling tool for infertile couples embarking on ART, who would like to gather information about their chances of success. However, the predictions can be used in conjunction with clinical knowledge for treatment planning as well as to develop patient awareness campaigns focusing on fertility preservation and the impact of female age on fertility. We studied the most important predictors for ART success using ~1,500 couples subjected to IVF/ICSI and PGT-A in Italy, Turkey, and Brazil. Additionally, we used robust methods for developing the validation model and comparing its fittings with the ART calculator. Furthermore, we computed the ART calculator predictive value and confirmed its clinical utility.

Limitations of our study include the inherent bias of a retrospective analysis and the fact that we were not able to assess all potentially relevant predictors. Baseline levels of FSH, sperm DNA fragmentation index, sperm morphology, and male BMI were excluded due to the inconsistent reporting by participating centers. Infertility duration, ethnicity, dietary patterns, smoking habits, alcohol consumption, history of previous pregnancy, and past PGT-A results were not taken into account as these predictors were not available in the dataset. Although these predictors may have an impact on ART success, their role on blastocyst euploidy remains to be elucidated. By contrast, the most important predictors for blastocyst euploidy according to the existing evidence were assessed, including female age, male factor, and ovarian reserve markers. While other validation studies exist for predictive models concerning live birth after a single or multiple IVF/ICSI cycles (64–66), no validation study like ours exists to predict the minimum number of mature oocytes needed to achieve at least one euploid blastocyst for transfer.

We acknowledge the variability in embryonic outcomes among centers and the intrinsic characteristics of different platforms used for comprehensive chromosomal screening, which could play a role in the accuracy of the calculator. Hence, we recommend caution when applying the ART calculator in other settings, as the coefficients of the fitted model might vary between centers.

### Future Research

Since our validation model was developed using retrospective data from ART centers, we will retest the model using a large prospective training cohort to provide even more accurate data in the future. Moreover, assessment of the ART calculator predictive value concerning (i) the oocyte genetic status by polar body analysis, and (ii) live birth rates are under consideration. We are currently sourcing suitable databases for conducting these studies.

## Conclusions

This study has validated a novel calculator to predict the minimum number of metaphase II oocytes required to achieve at least one euploid blastocyst in the general population of infertile patients undergoing IVF/ICSI. The ART calculator may be used as a point-of-care clinical toll for counseling and treatment planning in IVF/ICSI treatments.

## Data Availability Statement

All datasets generated for this study are included in the articles/Supplementary Material.

## Ethics Statement

The ethics committees of Instituto Investiga, Campinas, Brazil (CAAE 64291417.0.0000.5599), Hacettepe University, Ankara, Turkey (KA-180069), and Clinica Valle Giulia, Rome, Italy have approved the study.

## Author Contributions

SE designed and coordinated the study, wrote the article, affirms that the manuscript is an honest, accurate, and transparent account of the study reported and that no important aspects of the study have been omitted. JC designed the study, carried out the statistical analyses, and helped with data interpretation. FB, İÖ, MP, AV, DC, and LR coordinated data collection. HY, FU, and CA helped with coordination and data interpretation. All authors contributed intellectually to the writing or revising the manuscript and approved the final version.

### Conflict of Interest

SE, FU, and CA are co-founders of the POSEIDON group. SE declares receipt of unrestricted research grants from Merck, and lecture fees from Merck, Besins, Gedeon-Richter, and Lilly. CA has received honoraria for lectures from Merck. HY declares receipt of payment for lectures from Merck and Ferring. FU and AV have received honoraria for lectures from MSD and Merck. The remaining authors declare that the research was conducted in the absence of any commercial or financial relationships that could be construed as a potential conflict of interest.

## Supplementary Material

Supplementary Table 1 Patient and treatment variables in dataset.

Supplementary Table 2 Distribution of missing values and analyzed predictors.

Supplementary Table 3 Demographics and treatment characteristics of included couples by Anatolia Center (Turkey).

Supplementary Table 4 Demographics and treatment characteristics of included couples by GENERA (Italy).

Supplementary Table 5 Demographics and treatment characteristics of included couples by ANDROFERT Center (Brazil).

Supplementary Table 6 Adaptive Lasso regression analysis for variable selection.

Supplementary Table 7 ART calculator model for prediction of the probability (p) of euploid blastocyst per mature (MII) oocyte.

Supplementary Table 8 Raw data.

Supplementary Figure 1 Negative binomial distribution. Distribution of the number of euploid blastocysts. The summary statistics and quartiles show the descriptive data concerning the distribution of number of euploid blastocysts among 1,464 infertile couples undergoing IVF/ICSI and PGT-A. The distribution of euploid blastocysts per patient followed a negative binomial. This is evidenced by the univariate statistical analysis, where a gamma-Poisson distribution fits very well to the sample distribution. The fitted density function is shown in red in the histogram. The graph depicts the probability plot whose linearity evidences the appropriateness of the negative binomial.

Supplementary Figure 2 Differences on the predicted probabilities by the ART calculator and validation model.

Supplementary Figure 3 Comparison of fittings by ART calculator and validation model. The plots show the blastocyst euploidy predicted probabilities (per MII oocyte) by the ART calculator and validation model according to study center (Brazil = Androfert; Turkey = Anatolia; Italy = GENERA). The blue and red lines indicate the probability curves of the ART calculator and the validation model, respectively.

Supplementary Figure 4 Distribution of female age on dataset.

Supplementary Figure 5 Distribution of differences concerning the predicted probabilities (difP) according to the ART calculator and validation model by study center. The graphs depict the group of patients with 35 years and older, which correspond to over 90% or the validation dataset (see also Supplementary Figure 4).

Supplementary Video The movie shows how the ART calculator can be used in an office-based setting. Pretreatment, clinicians should input the patient age and the sperm source to be used for IVF/ICSI. If the option “Testicle” is marked, then the type of azoospermia should be also defined. The probability of success is set by the user and indicates the chance of having ≥1 euploid blastocyst when the predicted number of mature oocytes is achieved. Its complement is the risk, that is, the chance of having no (zero) euploid blastocysts when the predicted number of oocytes is achieved. Once the button “calculate” is pressed, a text box will pop-up on the right side of the screen, indicating the predicted minimum number of mature oocytes needed for obtaining at least one euploid blastocyst, with its 95% confidence interval. Posttreatment, i.e., when fewer than the predicted number of mature oocytes are obtained after one or more oocyte retrieval cycles. Clinicians should input the pretreatment information and the actual number of mature oocytes collected or accumulated. The probability of success is set by the user; it reflects the chance that the estimation is correct given the number of oocytes input. Once the button “calculate” is pressed, a text box will pop-up on the right side of the screen, indicating the predicted probability of achieving ≥1 euploid blastocyst with the number of mature oocytes available.
