# Supplementary material for: Validation of ART Calculator for Predicting the Number of Metaphase II Oocytes Required for Obtaining at Least One Euploid Blastocyst for Transfer in Couples Undergoing in vitro Fertilization/Intracytoplasmic Sperm Injection
Source: Front Endocrinol (Lausanne). 2020 Jan 24;10:917. doi: 10.3389/fendo.2019.00917 (PMC6992582; doi:10.3389/fendo.2019.00917)
Supplement: Supplementary Table 1 — Patient and treatment variables in dataset. [file Table_1.docx]

**Supplementary Table 1**. Patient and treatment variables in dataset

|  | **Type of variable (description)** |
| --- | --- |
| **A. Patient characteristics** | |
| Female age | Numerical/continuous (years) |
| Male age | Numerical/continuous (years) |
| Female body mass index | Numerical/continuous (kg/m^2^) |
| Male body mass index | Numerical/continuous (kg/m^2^) |
| Infertility duration | Numerical/continuous (years) |
| Infertility factor | Categorical/nominal (male factor, female factor, >1 type, unexplained) |
| Female infertility etiology | Categorical/nominal (endometriosis, endocrine/anovulatory, anatomic/tubal, unexplained, other, >1 type) |
| Basal FSH levels | Numerical/continuous (mUI/ml) |
| Ovarian reserve marker  *Antral follicle count*  *Anti-Müllerian hormone* | Numerical/continuous (n)  Numerical/continuous (ng/ml) |
| Poor ovarian reserve | Categorical/nominal (yes, no, undefined) |
| Associated male factor | Categorical/nominal (yes, no, undefined) |
| Sperm count | Numerical/continuous (x10^6^/ml) |
| Sperm motility (total) | Numerical/continuous (%) |
| Sperm morphology | Numerical/continuous (%) |
| Sperm DNA fragmentation | Numerical/continuous (%) |
| Presence of azoospermia | Categorical/nominal (yes, no, undefined) |
| Type of azoospermia | Categorical/nominal (obstructive, non-obstructive) |
| **B. Treatment characteristics** | |
| Type of ovarian stimulation | Categorical/nominal (conventional, minimal) |
| Type of gonadotropin used in conventional ovarian stimulation | Categorical/nominal (rFSH, rFSH+rLH) |
| Gonadotropin dose (total) | Numerical/continuous (IU) |
| Sperm source for ICSI | Categorical/nominal (ejaculate, epididymis, testicle) |
| Sperm status for ICSI | Categorical/nominal (fresh, cryopreserved) |
| Ejaculated sperm for ICSI | Categorical/nominal (homologous/abnormal, homologous/normal, heterologous) |
| Oocyte status | Categorical/nominal (fresh, vitrified-warmed) |
| **C. Outcome variables** | |
| No. oocytes retrieved | Numerical/continuous (n) |
| No. MII oocytes retrieved | Numerical/continuous (n) |
| No. 2PN zygotes | Numerical/continuous (n) |
| No. blastocysts | Numerical/continuous (n) |
| PGT-A result | Categorical/nominal (euploid, aneuploid) |
| No. euploid blastocysts | Numerical/continuous (n) |

FSH: follicle-stimulating hormone; ICSI: intracytoplasmic sperm injection; IU: international units; PGT-A: preimplantation genetic screening for aneuploidy; POR: poor ovarian reserve defined according to the POSEIDON criteria, namely, antral follicle count (AFC) <5 and/or anti-Müllerian hormone <1.2 ng/m; rFSH: recombinant FSH; rLH: recombinant LH; 2PN: two pronuclei; MII: metaphase II
