# Supplementary material for: Validation of ART Calculator for Predicting the Number of Metaphase II Oocytes Required for Obtaining at Least One Euploid Blastocyst for Transfer in Couples Undergoing in vitro Fertilization/Intracytoplasmic Sperm Injection
Source: Front Endocrinol (Lausanne). 2020 Jan 24;10:917. doi: 10.3389/fendo.2019.00917 (PMC6992582; doi:10.3389/fendo.2019.00917)
Supplement: Supplementary Table 2 — Distribution of missing values and analyzed predictors. [file Table_2.docx]

**Supplementary Table 2.** Distribution of missing values and analyzed predictors

| **Variable** | **N** | **N Missing** | **% Missing** | **Predictor** | **N included in analysis** |
| --- | --- | --- | --- | --- | --- |
| Age; Female | 1464 | 0 | 0.0 | Included | 1464 |
| Age; Male | 1464 | 0 | 0.0 | Included | 1464 |
| BMI; Female | 1464 | 0 | 0.0 | Included | 1464 |
| BMI; Male | 333 | 1131 | 77.2 | Excluded | NA |
| Ovarian reserve marker  *AMH*  *AFC* | 1464  1287  1464 | 0  177  0 | 0.0  12.1  0.0 | Included | 1464 |
| Baseline FSH levels | 408 | 1056 | 72.1 | Excluded | NA |
| Infertility factor | 1464 | 0 | 0.0 | Included | 1464 |
| Female infertility etiology | 1464 | 0 | 0.0 | Included | 1464 |
| Presence of azoospermia (yes/no) | 1464 | 0 | 0.0 | Included | 1464 |
| Type of azoospermia | 69† | 0 | 0.0 | Included | 69 |
| POR associated (yes/no) | 1464 | 0 | 0.0 | Included | 1464 |
| Male factor associated (yes/no) | 1464 | 0 | 0.0 | Included | 1464 |
| Sperm count (M/ml) | 1464 | 0 | 0.0 | Included | 1464 |
| Sperm motility (%; total) | 1395* | 0 | 0.0 | Included | 1395 |
| Sperm morphology (%) | 797 | 498 | 38.4 | Excluded | NA |
| Sperm DNA fragmentation (%) | 179 | 1285 | 87.8 | Excluded | NA |
| Type of gonadotropin | 1464 | 0 | 0.0 | Included | 1464 |
| Type of ovarian stimulation | 1464 | 0 | 0.0 | Included | 1464 |
| Gonadotropin dose | 1464 | 0 | 0.0 | Included | 1464 |
| Sperm status^1^ | 1464 | 0 | 0,0 | Included | 1464 |
| Oocyte status^2^ | 1464 | 0 | 0.0 | Included | 1464 |
| Source of sperm for ICSI^3^ | 1464 | 0 | 0.0 | Included | 1464 |
| Ejaculated sperm for ICSI^4^ | 1464 | 0 | 0.0 | Included | 1464 |
| Collected oocytes | 1464 | 0 | 0.0 | NA | NA |
| MII oocytes | 1464 | 0 | 0.0 | NA | NA |
| 2PN zygotes | 1464 | 0 | 0.0 | NA | NA |
| Blastocysts | 1464 | 0 | 0,0 | NA | NA |
| Euploid blastocysts | 1464 | 0 | 0.0 | NA | NA |
| Aneuploid blastocysts | 1464 | 0 | 0.0 | NA | NA |

BMI: body mass index; POR: poor ovarian reserve; NA: not applicable;

†A total of 69 azoospermia cases, in which surgically retrieved sperm were used for injections, were identified in the dataset; these cases were further categorized according to type (obstructive vs. non-obstructive azoospermia) and all records had complete data; the remaining 1395 cases had ejaculated sperm used for injections;

*Cases involving azoospermia were not computed;

^1^Fresh, frozen-thawed;

^2^Fresh, vitrified-warmed;

^3^Ejaculate, epididymis, testicle;

^4^Homologous/abnormal, homologous/normal, heterologous.
