# Supplementary material for: Validation of ART Calculator for Predicting the Number of Metaphase II Oocytes Required for Obtaining at Least One Euploid Blastocyst for Transfer in Couples Undergoing in vitro Fertilization/Intracytoplasmic Sperm Injection
Source: Front Endocrinol (Lausanne). 2020 Jan 24;10:917. doi: 10.3389/fendo.2019.00917 (PMC6992582; doi:10.3389/fendo.2019.00917)
Supplement: Supplementary Table 3 — Demographics and treatment characteristics of included couples by Anatolia Center (Turkey). [file Table_3.docx]

**Supplementary Table 3**. Demographics and treatment characteristics of included couples by Anatolia Center (Turkey)

| **Characteristics** | **N** | **Median** | **95% CI** |
| --- | --- | --- | --- |
| Female age (years) | 620 | 40.0 | 34.0-45.0 |
| Male age (years) | 620 | 42.0 | 33.0-51.5 |
| BMI, female (kg/m^2^) | 620 | 25.0 | 20.0-35.2 |
| BMI, male (kg/m^2^) | 0 | - | - |
| Infertility factor, N (%)  *Male factor*  *Unexplained*  *Endometriosis*  *Endocrine/Anovulatory*  *Anatomic/Tubal*  *>1 type* | 93 (15.0)  236 (38.1)  26 (4.2)  42 (6.8)  14 (2.2)  209 (33.7) | -  -  -  -  -  - | -  -  -  -  -  - |
| Baseline FSH (UI/mL) | 0 | - | - |
| Ovarian reserve marker  *AFC (n)*  *AMH (ng/mL)* | 620  508 | 7.0  1.0 | 2-20  0.1-5.0 |
| Semen parameters  *Sperm count (M/mL)*  *Total motility (%)*  *Sperm morphology (%)*  *DFI (%)* | 620  598  0  0 | 39.0  55.0  -  - | 1.0-125.0  24.0-77.0  -  - |
| Azoospermia; N (%)  *Non-obstructive; N (%)*  *Obstructive; N (%)* | 22 (3.5)  16 (2.6)  6 (0.9) | -  -  - | -  - - |
| POR associated, N (%) | 221 (35.6) | - | - |
| Male factor associated (%) | 149 (24.0) | - | - |
| Type of ovarian stimulation  *Conventional ovarian stimulation; N (%):*  *Minimal stimulation, N (%)* | 620 (100.0)  0 (0.0) | -  - | -  - |
| Type of gonadotropin; N (%)  *rFSH monotherapy*  *rFSH+rLH*  *rFSH+hMG*  *hMG alone*  *None* | 132 (21.3)  0 (0.0)  481 (77.6)  7 (1.1)  0 (0.0) | -  -  -  -  - | -  -  -  -  - |
| Total gonadotropin dose (IU) | 620 | 3150.0 | 1200.0-5850.0 |
| Sperm source for ICSI; N (%)  *Ejaculate*  *Epididymis*  *Testicle* | 598 (96.5)  4 (0.6)  18 (2.9) | -  -  - | -  -  - |
| Ejaculated sperm; N (%)  *Homologous; normal*  *Homologous; abnormal*  *Heterologous* | 480 (80.2)  118 (19.7)  0 (0.0) | -  -  - | -  -  - |
| Gamete status for ICSI; N (%)  *Fresh, sperm [S] + oocyte [O]*  *Cryopreserved, [S + O]*  *Combined, fresh [S] + vitrified-warmed [O]*  *Combined, frozen-thawed [S] + fresh [O]* | 617 (99.5)  0 (0.0)  0 (0.0)  3 (0.5) | -  -  -  - | -  -  -  - |
| Oocyte and embryo parameters  *No. Oocytes retrieved*  *No. Mature (MII) oocytes*  *No. Fertilized oocytes (2PN)*  *No. Blastocysts*  *No. Euploid blastocysts* | 620 | 6.0  5.0  3.0  1.0  0.0 | 1.0-17.0  1.0-14.0  0.0-11.0  0.0-5.5  0.0-3.0 |

BMI: body mass index; AFC: antral follicle count; AMH: anti-Müllerian hormone; DFI: Sperm DNA fragmentation index; FSH: follicle stimulating hormone; POR: poor ovarian reserve according to POSEIDON criteria; 2PN: two pronuclei zygote; MII: metaphase II
