# Supplementary material for: Validation of ART Calculator for Predicting the Number of Metaphase II Oocytes Required for Obtaining at Least One Euploid Blastocyst for Transfer in Couples Undergoing in vitro Fertilization/Intracytoplasmic Sperm Injection
Source: Front Endocrinol (Lausanne). 2020 Jan 24;10:917. doi: 10.3389/fendo.2019.00917 (PMC6992582; doi:10.3389/fendo.2019.00917)
Supplement: Supplementary Table 4 — Demographics and treatment characteristics of included couples by GENERA (Italy). [file Table_4.docx]

**Supplementary Table 4**. Demographics and treatment characteristics of included couples by GENERA (Italy)

| **Characteristics** | **N** | **Median** | **95% CI** |
| --- | --- | --- | --- |
| Female age (years) | 587 | 39.5 | 33.0-44.0 |
| Male age (years) | 587 | 41.0 | 33.0-51.0 |
| BMI, female (kg/m^2^) | 587 | 20.8 | 18.0-27.7 |
| BMI, male (kg/m^2^) | 93 | 24.4 | 21.4-31.2 |
| Infertility factor, N (%)  *Male factor*  *Unexplained*  *Endometriosis*  *Endocrine/Anovulatory*  *Anatomic/Tubal*  *>1 type* | 105 (17.9)  283 (48.2)  28 (4.8)  48 (8.2)  19 (3.2)  104 (17.7) | -  -  -  -  -  - | -  -  -  -  -  - |
| Baseline FSH (UI/mL) | 181 | 7.7 | 4.9-15.0 |
| Ovarian reserve marker  *AFC (n)*  *AMH (ng/mL)* | 587  587  522 | 9.0  1.7 | 2-29  0.3-8.0 |
| Semen parameters  *Sperm count (M/mL)*  *Total motility (%)*  *Sperm morphology (%)*  *DFI (%)* | 587  570  570  0 | 28.0  50.0  4.0  - | 1.0-80.0  10.0-65.0  1.0-9.0  - |
| Azoospermia; N (%)  *Non-obstructive; N (%)*  *Obstructive; N (%)* | 17 (2.9)  9 (1.5)  8 (1.4) | -  -  - | -  - - |
| POR associated, N (%) | 125 (21.3) | - | - |
| Male factor associated (%) | 133 (22.7) | - | - |
| Type of ovarian stimulation  *Conventional ovarian stimulation; N (%):*  *Minimal stimulation, N (%)* | 556 (94.7)  31 (5.3) | -  - | -  - |
| Type of gonadotropin; N (%)  *rFSH monotherapy*  *rFSH+rLH*  *rFSH+hMG*  *hMG alone*  *None* | 308 (52.5)  119 (20.3)  51 (8.7)  89 (15.1)  20 (3.4) | -  -  -  -  - | -  -  -  -  - |
| Total gonadotropin dose (IU) | 587 | 2700.0 | 1120.0-4150.0 |
| Sperm source for ICSI; N (%)  *Ejaculate*  *Epididymis*  *Testicle* | 570 (97.1)  8 (1.4)  9 (1.5) | -  -  - | -  -  - |
| Ejaculated sperm; N (%)  *Homologous; normal*  *Homologous; abnormal*  *Heterologous* | 251 (44.0)  317 (55.6)  2 (0.4) | -  -  - | -  -  - |
| Gamete status for ICSI; N (%)  *Fresh, sperm [S] + oocyte [O]*  *Cryopreserved, [S + O]*  *Combined, fresh [S] + vitrified-warmed [O]*  *Combined, frozen-thawed [S] + fresh [O]* | 547 (93.2)  0 (0.0)  2 (0.3)  38 (6.5) | -  -  -  - | -  -  -  - |
| Oocyte and embryo parameters  *No. Oocytes retrieved*  *No. Mature (MII) oocytes*  *No. Fertilized oocytes (2PN)*  *No. Blastocysts*  *No. Euploid blastocysts* | 587 | 10.0  7.0  5.0  2.0  1.0 | 2.0-25.0  1.0-17.0  1.0-13.0  0.0-7.0  0.0-5.0 |

BMI: body mass index; AFC: antral follicle count; AMH: anti-Müllerian hormone; DFI: Sperm DNA fragmentation index; FSH: follicle stimulating hormone; POR: poor ovarian reserve according to POSEIDON criteria; 2PN: two pronuclei zygote; MII: metaphase II
