# Supplementary material for: Validation of ART Calculator for Predicting the Number of Metaphase II Oocytes Required for Obtaining at Least One Euploid Blastocyst for Transfer in Couples Undergoing in vitro Fertilization/Intracytoplasmic Sperm Injection
Source: Front Endocrinol (Lausanne). 2020 Jan 24;10:917. doi: 10.3389/fendo.2019.00917 (PMC6992582; doi:10.3389/fendo.2019.00917)
Supplement: Supplementary Table 5 — Demographics and treatment characteristics of included couples by ANDROFERT Center (Brazil). [file Table_5.docx]

**Supplementary Table 5**. Demographics and treatment characteristics of included couples by ANDROFERT Center (Brazil)

| **Characteristics** | **N** | **Median** | **95% CI** |
| --- | --- | --- | --- |
| Female age (years) | 257 | 39.0 | 32.0-44.0 |
| Male age (years) | 257 | 41.0 | 34.0-55.0 |
| BMI, female (kg/m^2^) | 257 | 23.8 | 19.3-35.6 |
| BMI, male (kg/m^2^) | 241 | 27.6 | 21.9-31.1 |
| Infertility factor, N (%)  *Male factor*  *Unexplained*  *Endometriosis*  *Endocrine/Anovulatory*  *Anatomic/Tubal*  *>1 type* | 54 (21.0)  25 (9.7)  4 (1.6)  16 (6.2)  22 (8.5)  136 (53.0) | -  -  -  -  -  - | -  -  -  -  -  - |
| Baseline FSH (UI/mL) | 227 | 7.8 | 4.5-14.5 |
| Ovarian reserve marker  *AFC (n)*  *AMH (ng/mL)* | 257  257  257 | 6.0  1.0 | 2-14  0.0-3.8 |
| Semen parameters  *Sperm count (M/mL)*  *Total motility (%)*  *Sperm morphology (%)*  *DFI (%)* | 257  227  227  179 | 19.0  66.0  2.0  19.0 | 0.0-94.8  32.0-81.0  0.0-7.0  7.0-50.0 |
| Azoospermia; N (%)  *Non-obstructive; N (%)*  *Obstructive; N (%)* | 30 (11.7)  10 (3.9)  20 (7.8) | -  -  - | -  - - |
| POR associated, N (%) | 136 (52.9) | - | - |
| Male factor associated (%) | 176 (68.5) | - | - |
| Type of ovarian stimulation  *Conventional ovarian stimulation; N (%):*  *Minimal stimulation, N (%)* | 190 (73.9)  67 (26.1) | -  - | -  - |
| Type of gonadotropin; N (%)  *rFSH monotherapy*  *rFSH+rLH*  *rFSH+hMG*  *hMG alone*  *None* | 73 (28.4)  177 (68.9)  6 (2.3)  1 (0.4)  0 (0.0) | -  -  -  -  - | -  -  -  -  - |
| Total gonadotropin dose (IU) | 257 | 3200.0 | 300.0-4950.0 |
| Sperm source for ICSI; N (%)  *Ejaculate*  *Epididymis*  *Testicle* | 190 (74.0)  5 (1.9)  62 (24.1) | -  -  - | -  -  - |
| Ejaculated sperm; N (%)  *Homologous; normal*  *Homologous; abnormal*  *Heterologous* | 30 (15.8)  152 (80.0)  8 (4.2) | -  -  - | -  -  - |
| Gamete status for ICSI; N (%)  *Fresh, sperm [S] + oocyte [O]*  *Cryopreserved, [S + O]*  *Combined, fresh [S] + vitrified-warmed [O]*  *Combined, frozen-thawed [S] + fresh [O]* | 224 (87.2)  0 (0.0)  5 (1.9)  28 (10.9) | -  -  -  - | -  -  -  - |
| Oocyte and embryo parameters  *No. Oocytes retrieved*  *No. Mature (MII) oocytes*  *No. Fertilized oocytes (2PN)*  *No. Blastocysts*  *No. Euploid blastocysts* | 257 | 7.0  5.0  3.0  1.0  0.0 | 210-24.0  1.0-18.0  0.0-13.0  0.0-6.0  0.0-3.0 |

BMI: body mass index; AFC: antral follicle count; AMH: anti-Müllerian hormone; DFI: Sperm DNA fragmentation index; FSH: follicle stimulating hormone; POR: poor ovarian reserve according to POSEIDON criteria; 2PN: two pronuclei zygote; MII: metaphase II
