# Supplementary material for: Validation of ART Calculator for Predicting the Number of Metaphase II Oocytes Required for Obtaining at Least One Euploid Blastocyst for Transfer in Couples Undergoing in vitro Fertilization/Intracytoplasmic Sperm Injection
Source: Front Endocrinol (Lausanne). 2020 Jan 24;10:917. doi: 10.3389/fendo.2019.00917 (PMC6992582; doi:10.3389/fendo.2019.00917)
Supplement: Supplementary Table 6 — Adaptive Lasso regression analysis for variable selection. [file Table_6.docx]

**Supplementary Table 6.** Adaptive Lasso regression analysis for variable selection

| *Term* | *Estimate* | *Standard error* | *Wald ChiSquare* | *Prob > ChiSquare* | *Lower 95%* | *Upper 95%* |
| --- | --- | --- | --- | --- | --- | --- |
| (Intercept) | 6.4361056 | 1.679 | 14.688 | 0.0001 | 3.144 | 9.727 |
| **Female Age** | -0.194763 | 0.043 | 20.050 | **<0.0001** | -0.280 | -0.109 |
| Male age | 0 | 0 | 0 | 1.000 | 0 | 0 |
| BMI, Female | 0 | 0 | 0 | 1.000 | 0 | 0 |
| Ovarian reserve marker [AMH-AFC] | 0 | 0 | 0 | 1.000 | 0 | 0 |
| Female infertility [Combined-Unexplained] | 0 | 0 | 0 | 1.000 | 0 | 0 |
| Female infertility [Female-Unexplained] | 0 | 0 | 0 | 1.000 | 0 | 0 |
| Female infertility [Male-Unexplained] | 0 | 0 | 0 | 1.000 | 0 | 0 |
| Female infertility [Anatomic-None] | 0 | 0 | 0 | 1.000 | 0 | 0 |
| Female infertility [Anatomic-Endocrine-None] | 0 | 0 | 0 | 1.000 | 0 | 0 |
| Female infertility [Anatomic-Endometriosis-None] | 0 | 0 | 0 | 1.000 | 0 | 0 |
| Female infertility [Endocrine-None] | 0 | 0 | 0 | 1.000 | 0 | 0 |
| Female infertility [Endometriosis-None] | 0 | 0 | 0 | 1.000 | 0 | 0 |
| Female infertility [Endocrine-Endometriosis-None] | 0 | 0 | 0 | 1.000 | 0 | 0 |
| POR associated | 0 | 0 | 0 | 1.000 | 0 | 0 |
| Male factor associated | 0 | 0 | 0 | 1.000 | 0 | 0 |
| Semen [homologous normal-homologous abnormal] | 0 | 0 | 0 | 1.000 | 0 | 0 |
| Semen [homologous normal-heterologous] | 0 | 0 | 0 | 1.000 | 0 | 0 |
| Semen [homologous abnormal-heterologous] | 0 | 0 | 0 | 1.000 | 0 | 0 |
| Sperm count | 0 | 0 | 0 | 1.000 | 0 | 0 |
| Sperm motility | 0 | 0 | 0 | 1.000 | 0 | 0 |
| Ovarian stimulation type [Conventional-Minimal/Natural] | 0 | 0 | 0 | 1.000 | 0 | 0 |
| Gonadotropins [rFSH+rLH-None] | 0 | 0 | 0 | 1.000 | 0 | 0 |
| Gonadotropins [rFSH alone-hMG] | 0 | 0 | 0 | 1.000 | 0 | 0 |
| Gonadotropins [rFSH alone-rFSH+rLH] | 0 | 0 | 0 | 1.000 | 0 | 0 |
| Gonadotropins [rFSH alone-None] | 0 | 0 | 0 | 1.000 | 0 | 0 |
| Gonadotropins [rFSH+rLH-hMG] | 0 | 0 | 0 | 1.000 | 0 | 0 |
| Gonadotropins [hMG-None] | 0 | 0 | 0 | 1.000 | 0 | 0 |
| Gonadotropins [rFSH or rFSH-rFSH+hMG] | 0 | 0 | 0 | 1.000 | 0 | 0 |
| Gonadotropin dose | 0 | 0 | 0 | 1.000 | 0 | 0 |
| Sperm status for ICSI [FRESH-FROZEN-THAWED] | 0 | 0 | 0 | 1.000 | 0 | 0 |
| Oocyte status [FRESH-VITRIFIED-WARMED] | 0 | 0 | 0 | 1.000 | 0 | 0 |
| **MII oocytes** | 0.0831113 | 0.014 | 34.540 | **<0.0001** | 0.055 | 0.110 |
| SpermSource | 0 | 0 | 0 | 1.000 | 0 | 0 |
| Dispersion | 7.5127e-6 | 0.654 | 1.32e-10 | 1.000 | -1.281 | 1.281 |
| *Statistics:*  Response: Euploid blastocysts  Distribution: negative binomial  Estimation method: Adaptive Lasso with validation column  Mean model link: Log  Dispersion model link: Identity  Number of rows: 1464  Sum of Frequencies: 157  -LogLikelihood: 135.783  Number of Parameters: 4  BIC: 291.791  AICc: 279.830  Generalized RSquare: 0.430  Lambda Penalty: 45.175 | | | | | | |
