# Supplementary material for: Validation of ART Calculator for Predicting the Number of Metaphase II Oocytes Required for Obtaining at Least One Euploid Blastocyst for Transfer in Couples Undergoing in vitro Fertilization/Intracytoplasmic Sperm Injection
Source: Front Endocrinol (Lausanne). 2020 Jan 24;10:917. doi: 10.3389/fendo.2019.00917 (PMC6992582; doi:10.3389/fendo.2019.00917)
Supplement: Supplementary Table 7 — ART calculator model for prediction of the probability (p) of euploid blastocyst per mature (MII) oocyte. [file Table_7.docx]

**Supplementary Table 7**. ART calculator model for prediction of the probability (p) of euploid blastocyst per mature (MII) oocyte

| *Equation*  *Y = a+b [Sperm ="Ejaculate"] +c [Sperm = Ejaculate](FemaleAge-38.9066) + d [Sperm=Testicular_NOA](FemaleAge-38.9066),*  *where* $p=\left( \frac{1}{1+e^{-y}} \right)$ | | | | |
| --- | --- | --- | --- | --- |
| *Term* | *Estimate* | *SE* | *Wald ChiSquare* | *Prob >ChiSquare* |
| (Intercept) | -2.6518 | 0.1174497 | 371.96 | <.0001 |
| spermSource [EJACULATE]:(ageFemale-37.9384) | -0.2045457 | 0.0269435 | 57.63 | <.0001 |
| spermSource [TESTICULAR_NOA]:(ageFemale-37.9384) | -0.1530924 | 0.0354465 | 18.65 | <.0001 |
| spermSource [Ejaculate] | 0.2231659 | 0.1174497 | 3.61 | 0.0574 |
|  |  |  |  |  |
| *Statistics:*  Response: euploid blastocyst given MII oocytes  Distribution: binomial  Estimation method: Nominal logistic fit  Mean model link: Logit  Area under the curve: 0.71589 |  |  |  |  |

The Nominal Logistic Fit was the final model with the best prediction of the probability of ≥1 euploid blastocyst per mature (MII) oocyte. The full equation is written at the top of the table. Each particular characteristic is displayed with an associated P value (*Prob >ChiSquare*) giving the indication of how much weight each variable will contribute to the predictive number of mature oocytes. a=intercept; b= spermSource [EJACULATE]; c**_v_**= spermSource [EJACULATE]:(ageFemale-37.9384), d = spermSource [NOA]:(ageFemale-37.9384); SE = standard error.

Reprint from Esteves et al. [17]
