# Supplementary figures and images for: Validation of ART Calculator for Predicting the Number of Metaphase II Oocytes Required for Obtaining at Least One Euploid Blastocyst for Transfer in Couples Undergoing in vitro Fertilization/Intracytoplasmic Sperm Injection
Source: Front Endocrinol (Lausanne). 2020 Jan 24;10:917. doi: 10.3389/fendo.2019.00917 (PMC6992582; doi:10.3389/fendo.2019.00917)

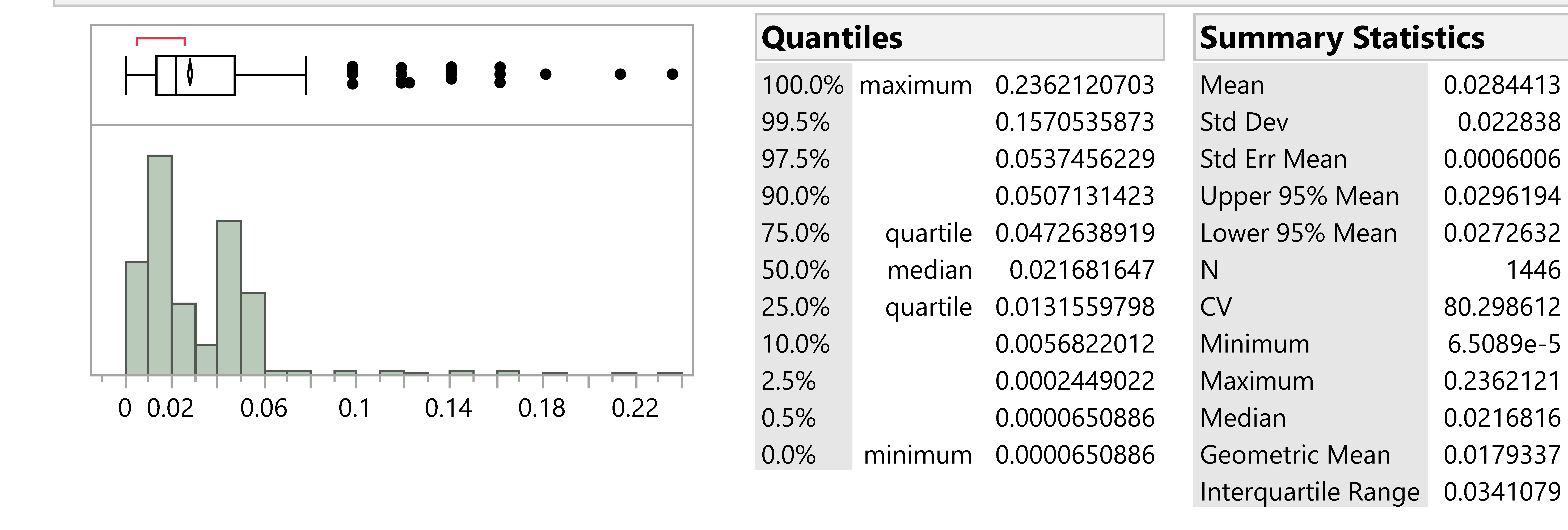

Supplement: Supplementary Figure 2 — Differences on the predicted probabilities by the ART calculator and validation model. [file Image_2.JPEG]

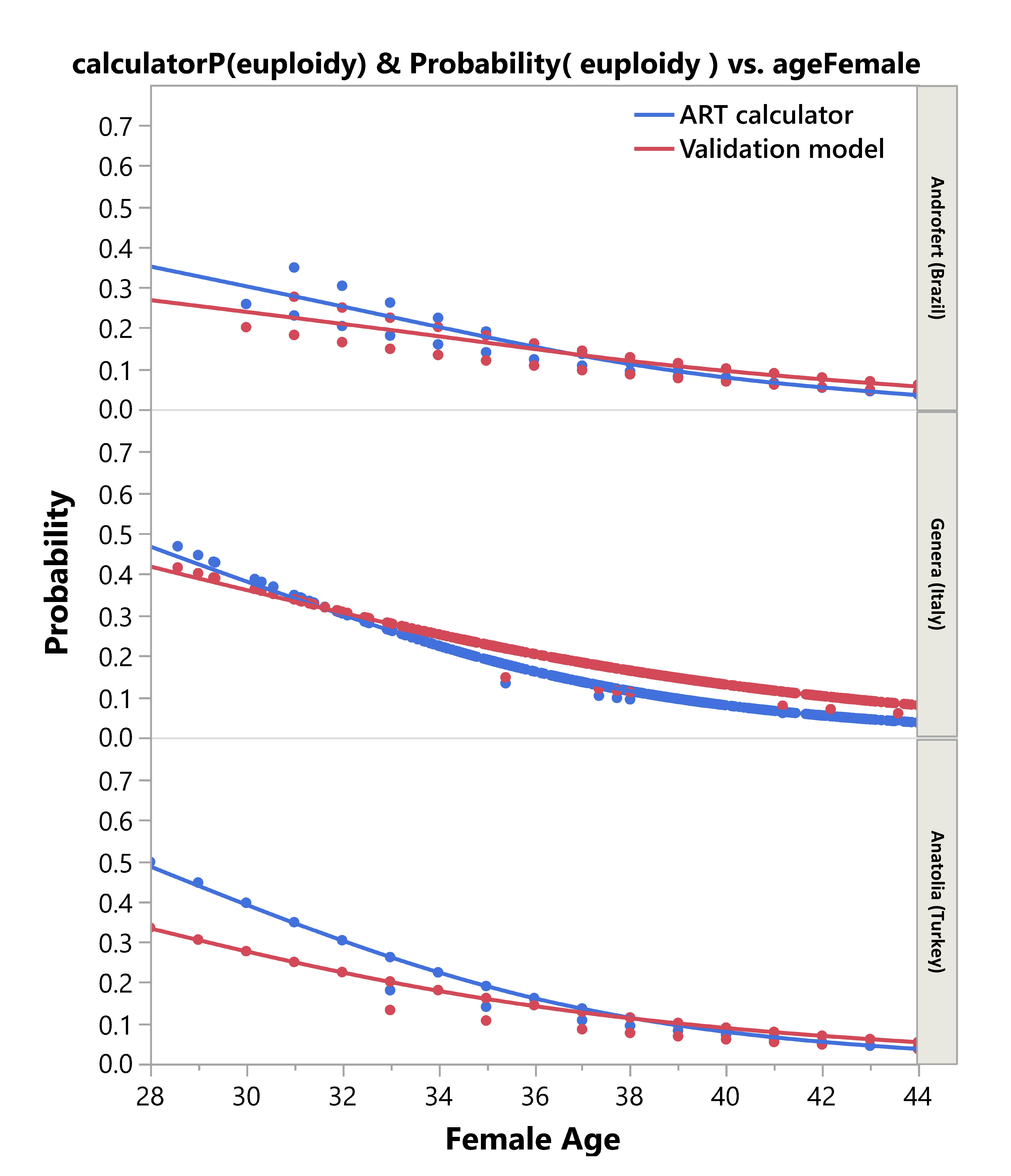

Supplement: Supplementary Figure 3 — Comparison of fittings by ART calculator and validation model. The plots show the blastocyst euploidy predicted probabilities (per MII oocyte) by the ART calculator and validation model according to study center (Brazil = Androfert; Turkey = Anatolia; Italy = GENERA). The blue and red lines indicate the probability curves of the ART calculator and the validation model, respectively. [file Image_3.JPEG]

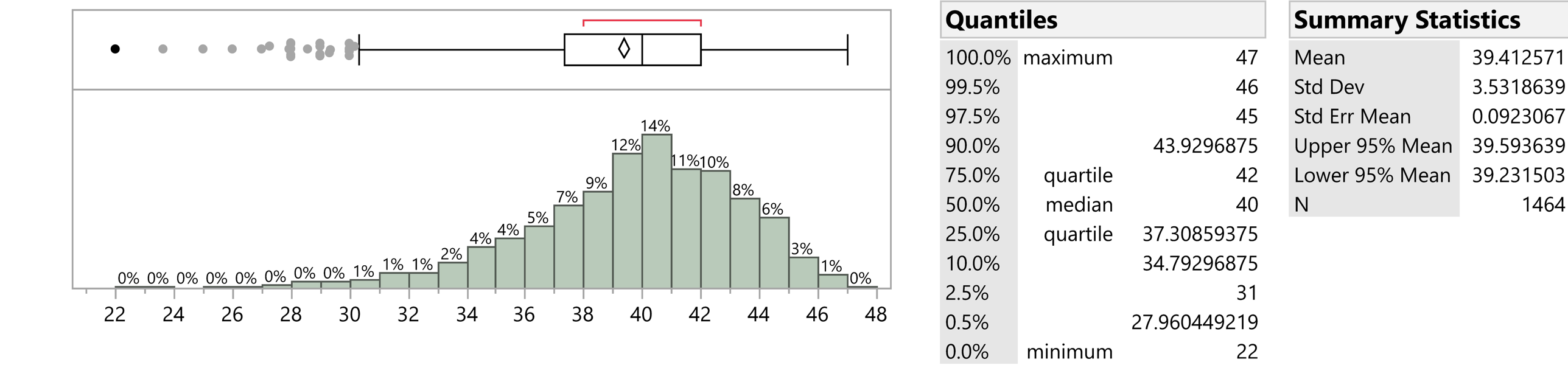

Supplement: Supplementary Figure 4 — Distribution of female age on dataset. [file Image_4.JPEG]
