# Supplementary material for: Validation of ART Calculator for Predicting the Number of Metaphase II Oocytes Required for Obtaining at Least One Euploid Blastocyst for Transfer in Couples Undergoing in vitro Fertilization/Intracytoplasmic Sperm Injection
Source: Front Endocrinol (Lausanne). 2020 Jan 24;10:917. doi: 10.3389/fendo.2019.00917 (PMC6992582; doi:10.3389/fendo.2019.00917)
Supplement: Supplementary file 15 [file Data_Sheet_1.docx]

**Supplementary data sheet.** Analysis of embryonic outcomes that might have influenced the probabilities of an MII oocyte turning into a euploid blastocyst in the study centers performing NGS (Anatolia and Androfert) and qPCR (Genera). The multiple comparison of means was performed using the Tukey-Kramer HSD (honestly significant difference) test, which is an exact alpha-level test if the sample sizes are the same, and conservative if the sample sizes are different. The graphs below show the distribution of 2PN fertilization rates (A) and blastulation rates (B) by study center. On the right-hand side, comparison circles plot provides a visual representation of group means comparisons. The circles are nested, thus indicating that the means are not significantly different. The threshold matrix shows the actual absolute difference in the means minus the HSD, which is the difference that would be significant. Pairs with a positive value are significantly different. The q* (appearing above the HSD Threshold Matrix table) is the quantile that is used to scale the HSDs. It has a computational role comparable to a Student’s t.

1. Fertilization rates


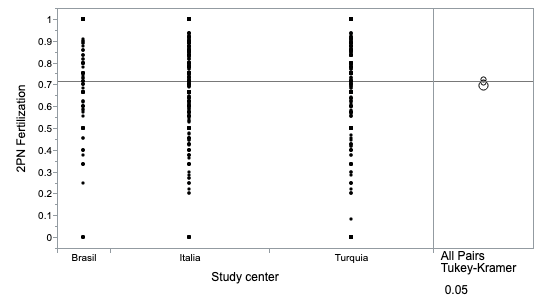


Means Comparisons

Comparisons for all pairs using Tukey-Kramer HSD

Confidence Quantile

| q* | Alpha |
| --- | --- |
| 2.34632 | 0.05 |

HSD Threshold Matrix

| Abs(Dif)-HSD | | | |
| --- | --- | --- | --- |
|  | Genera | Anatolia | Androfert |
| Genera | -0.03711 | -0.02053 | -0.02591 |
| Anatolia | -0.02053 | -0.03660 | -0.04206 |
| Androfert | -0.02591 | -0.04206 | -0.06468 |

Positive values show pairs of means that are significantly different.

Connecting Letters Report

| Level |  |  |  |  |  |  |  |  |  |  |  |  |  |  |  | Mean |
| --- | --- | --- | --- | --- | --- | --- | --- | --- | --- | --- | --- | --- | --- | --- | --- | --- |
| Genera | A |  |  |  |  |  |  |  |  |  |  |  |  |  |  | 0.72608797 |
| Anatolia | A |  |  |  |  |  |  |  |  |  |  |  |  |  |  | 0.70976215 |
| Androfert | A |  |  |  |  |  |  |  |  |  |  |  |  |  |  | 0.69926643 |

Levels not connected by same letter are significantly different.

Ordered Differences Report

| Level | - Level | Difference | Std Err Dif | Lower CL | Upper CL | p-Value |  |
| --- | --- | --- | --- | --- | --- | --- | --- |
| Genera | Androfert | 0.0268215 | 0.0224741 | -0.025910 | 0.0795530 | 0.4573 |  |
| Genera | Anatolia | 0.0163258 | 0.0157090 | -0.020533 | 0.0531842 | 0.5522 |  |
| Anatolia | Androfert | 0.0104957 | 0.0223977 | -0.042057 | 0.0630479 | 0.8860 |  |

B. Blastulation rates


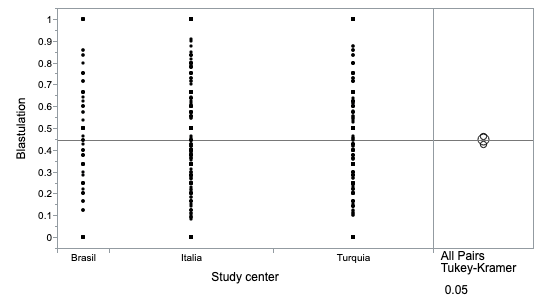


Means Comparisons

Comparisons for all pairs using Tukey-Kramer HSD

Confidence Quantile

| q* | Alpha |
| --- | --- |
| 2.34649 | 0.05 |

HSD Threshold Matrix

| Abs(Dif)-HSD | | | |
| --- | --- | --- | --- |
|  | Genera | Androfert | Anatolia |
| Genera | -0.04393 | -0.04949 | -0.00841 |
| Androfert | -0.04949 | -0.07759 | -0.04102 |
| Anatolia | -0.00841 | -0.04102 | -0.04433 |

Positive values show pairs of means that are significantly different.

Connecting Letters Report

| Level |  |  |  |  |  |  |  |  |  |  |  |  |  |  |  | Mean |
| --- | --- | --- | --- | --- | --- | --- | --- | --- | --- | --- | --- | --- | --- | --- | --- | --- |
| Genera | A |  |  |  |  |  |  |  |  |  |  |  |  |  |  | 0.46116610 |
| Androfert | A |  |  |  |  |  |  |  |  |  |  |  |  |  |  | 0.44761224 |
| Anatolia | A |  |  |  |  |  |  |  |  |  |  |  |  |  |  | 0.42544492 |

Levels not connected by same letter are significantly different.

Ordered Differences Report

| Level | - Level | Difference | Std Err Dif | Lower CL | Upper CL | p-Value |  |
| --- | --- | --- | --- | --- | --- | --- | --- |
| Genera | Anatolia | 0.0357212 | 0.0188070 | -0.008409 | 0.0798516 | 0.1393 |  |
| Androfert | Anatolia | 0.0221673 | 0.0269288 | -0.041021 | 0.0853556 | 0.6887 |  |
| Genera | Androfert | 0.0135539 | 0.0268680 | -0.049492 | 0.0765995 | 0.8692 |  |
